# Piperlongumine inhibits migration and proliferation of castration-resistant prostate cancer cells via triggering persistent DNA damage

**DOI:** 10.1186/s12906-021-03369-0

**Published:** 2021-07-06

**Authors:** Ding-fang Zhang, Zhi-chun Yang, Jian-qiang Chen, Xiang-xiang Jin, Yin-da Qiu, Xiao-jing Chen, Hong-yi Shi, Zhi-guo Liu, Min-shan Wang, Guang Liang, Xiao-hui Zheng

**Affiliations:** 1 Chemical Biology Research Center, School of Pharmaceutical Sciences, Wenzhou Medical University, Wenzhou, 325035 Zhejiang People’s Republic of China; 2 The Fifth Affiliated Hospital of Wenzhou Medical University, Affiliated Lishui Hospital of Zhejiang University, The Central Hospital of Zhejiang Lishui, Lishui, 323000 Zhejiang People’s Republic of China; 3 The Affiliated Xiangshan Hospital, Wenzhou Medical University, Ningbo, 315000 Zhejiang China; 4 Hospital of Chinese Medicine of Haishu District, Ningbo, 315000 Zhejiang China

**Keywords:** Castration-resistant prostate cancer, Piperlongumine, Cancer migration, DNA damage, DNA repair, DNA damage response

## Abstract

**Background:**

Metastatic castration-resistant prostate cancer (CRPC) is the leading cause of death among men diagnosed with prostate cancer. Piperlongumine (PL) is a novel potential anticancer agent that has been demonstrated to exhibit anticancer efficacy against prostate cancer cells. However, the effects of PL on DNA damage and repair against CRPC have remained unclear. The aim of this study was to further explore the anticancer activity and mechanisms of action of PL against CRPC in terms of DNA damage and repair processes.

**Methods:**

The effect of PL on CRPC was evaluated by MTT assay, long-term cell proliferation, reactive oxygen species assay, western blot assay, flow cytometry assay (annexin V/PI staining), β-gal staining assay and DAPI staining assay. The capacity of PL to inhibit the invasion and migration of CRPC cells was assessed by scratch-wound assay, cell adhesion assay, transwell assay and immunofluorescence (IF) assay. The effect of PL on DNA damage and repair was determined via IF assay and comet assay.

**Results:**

The results showed that PL exhibited stronger anticancer activity against CRPC compared to that of taxol, cisplatin (DDP), doxorubicin (Dox), or 5-Fluorouracil (5-FU), with fewer side effects in normal cells. Importantly, PL treatment significantly decreased cell adhesion to the extracellular matrix and inhibited the migration of CRPC cells through affecting the expression and distribution of focal adhesion kinase (FAK), leading to concentration-dependent inhibition of CRPC cell proliferation and concomitantly increased cell death. Moreover, PL treatment triggered persistent DNA damage and provoked strong DNA damage responses in CRPC cells.

**Conclusion:**

Collectively, our findings demonstrate that PL potently inhibited proliferation, migration, and invasion of CRPC cells and that these potent anticancer effects were potentially achieved via triggering persistent DNA damage in CRPC cells.

**Supplementary Information:**

The online version contains supplementary material available at 10.1186/s12906-021-03369-0.

## Background

Castration-resistant prostate cancer (CRPC), which develops from prostate cancer but is resistant to androgen-deprivation therapy [1], is the leading cause of death in prostate cancer [2, 3]. The high mortality rate of CRPC is mainly due to metastases to the bone and/or brain [4, 5]. Currently, mainstay treatments for CRPC include surgery, chemotherapy, radiotherapy, and immunotherapy [1, 6]. Among them, chemotherapy is the first choice for oncologists in terms of treating metastatic CRPC. The majority of approved chemotherapeutic drugs kill cancer cells by introducing a DNA damage response [7]. Cancer cells are endowed with a similar or even stronger innate DNA-repair capacity compared to that of normal cells [8], which can limit the effectiveness of approved agents in treating cancer and/or may lead to chemotherapy failure [9]. With these concerns, an inexpensive reagent that enhances DNA damage and inhibits DNA repair may have a potential advantage as a CRPC therapeutic drug, as identification of drugs that target DNA damage with low side effects in normal cells remains a challenge in CRPC chemotherapy.

Piperlongumine (PL) is a natural antibacterial compound that is readily available, is inexpensive, and has long been used in Chinese herbal and Indian Ayurvedic medicine [10]. Recently, PL has received increased attention from researchers due to its anticancer effects [11, 12]. Mechanistic investigations have revealed that PL achieves anticancer effects via a reactive oxygen species (ROS)-dependent pathway [13] and modulates related signaling pathways, including MAPK, NF-κB, and STAT3 pathways [14–20]. In the last decade, PL has been demonstrated to exhibit anticancer efficacies as well as sensitize the anticancer activities of chemotherapeutic drugs (e.g., doxorubicin) against prostate cancer cells [14, 21, 22]. However, the effects of PL on DNA damage and repair in CRPC cells have remained unclear. Hence, our present study investigated and identified the effects of PL on DNA damage and repair in CRPC PC3 and DU145 cells, which are derived from bone and brain metastasis of CRPC.

## Methods

### Cell cultures and reagents

CRPC cells (PC3 and DU145), the human normal prostatic stromal myofibroblast cell line (WPMY-1), and the human normal hepatic cell line (LO2) were purchased from the Cell Resource Center of Peking Union Medical College. Cells were maintained at 37 °C with 5% CO_2_ in Dulbecco’s modified eagle’s medium (DMEM; Gibco) that was supplemented with 10% fetal calf serum (PPA-GE, Marlborough, MA), as well as 100 U/mL of penicillin and streptomycin (HyClone-GE, Marlborough, MA). N-acetyl-L-cysteine (NAC) was purchased from Beyotime Biotech (China). PL was obtained from Sigma-Aldrich (Item Number: SML0221), with a purity of ≥97%.

### Determination of cell viability

The cell viabilities of PC3, DU145, WPMY-1, and LO2 cells were assessed via 3-(4,5-dimethylthiazol-2-yl)-2,5-diphenyltetrazolium bromide (MTT) assays [23]. Cells (4 × 10^3^ cells/well in 96-well plates) were incubated at 37 °C with or without PL treatment for 48 h, after which MTT (0.5 mg/mL) was added at 20 μL/well for another 4 h. The reaction product, formazan, was dissolved in 100 μL of DMSO after discarding the culture medium. Cell viability was determined by reading the absorbance at 560 nm by a spectrophotometer (DTX880, Beckman Coulter, CA, USA).

### Cell adhesion assays

Cell adhesion assays were performed as described previously [24]. A 96-well plate was coated with 50 μL of human fibronectin (2.5 μg/mL) in 1 × PBS (Millipore, CA) at 4 °C overnight. Cells were seeded into a 96-well plate at a density of 4 × 10^4^ cells/well and were cultured for another 1 h at 37 °C in an incubator with 5% CO_2_. Cells were then rinsed three times with 10% formalin and stained with crystal violet for 5 min at room temperature. After three washes with double-distilled H_2_O (ddH_2_O), stained cells were dissolved in 100 μL of acetic acid (33%). The absorbance at 560 nm was detected by a Synergy H1 Multi-Mode Reader (BioTek). The relative number of cells attached to the extracellular matrix was calculated using the following equation: mean optical density (OD) of treated cells/mean OD of control cells. Cells treated with vehicle (0.01% DMSO) were used as a control.

### Transwell assays

Transwell assays were performed as described previously [24] and carried out according to a purchased transwell kit (Corning Costar, NY) following the manufacturer’s instructions. Briefly, cells were pretreated with different concentrations of PL for 48 h and were then reseeded into transwell permeable support (insert) pre-equilibrated with serum-free DMEM medium. For each group, 1 × 10^5^ cells/insert were seeded and incubated in 100 μL of serum-free DMEM medium. The insert was then placed in a 24-well plate containing 600 μL of DMEM medium with 10% FBS. After 24 h of culturing, cells on the upper surface of the insert were removed with cotton-tipped swabs. Then, the cells on the backside surface of the insert were fixed with 10% formalin, stained with crystal violet for 5 min at room temperature, and washed three times with ddH_2_O. Stained cells were dissolved in 500 μL of acetic acid (33%), and their absorbances were detected at 560 nm by a spectrophotometer (DTX880, Beckman Coulter, CA, USA).

### Scratch-wound assays

Cell scratch-wound assays were performed as described previously [24]. The cells were seeded in a six-well plate at a density of 3 × 10^5^ cells/well and were cultured in medium containing PL or 0.01% DMSO for 48 h. A denuded area was created across the diameter of the dish by a yellow tip. The cells were washed with PBS and then incubated in a serum-free medium. Phase-contrast images were acquired at the indicated times of incubation. Images were analyzed with Axiovision Rel.4.8 software. The percentage of areas covered by migrated cells (i.e., wound recovery) was calculated. Three independent experiments were carried out for quantification.

### Annexin V/PI apoptotic assays

The Annexin V/PI method was applied for apoptotic assays (Sigma) following the manufacturer’s instructions. Cells were seeded in 10cm^2^ dishes at a density of 5.0 × 10^5^ cells per dish and were incubated at 37 °C overnight. PL at the indicated concentration was then added into the medium. After long-term cell proliferation assays or scratch-wound assays, cells were harvested for anexin V/PI apoptotic assays. These assays were performed following the protocol provided by the Annexin V/PI Apoptosis Kit (Sigma) and were assessed via a flow cytometer (BD FACS Calibur, BD Biosciences).

### Senescence-associated beta-galactosidase (SA-β gal) activity

A beta-galactosidase (SA-β gal) staining kit was obtained from Sigma. The assay was performed following the manufacturer’s instructions. In brief, cells were washed once with PBS and fixed with stationary liquid provided in the kit at room temperature for 15 min. Next, the cells were incubated overnight at 37 °C in the dark with 1 mL of a working solution containing 0.05 mg/ml of 5-bromo-4-chloro-3-indolyl-b-d-galactopyranoside (X-gal). Subsequently, the cells were observed under a normal light microscope (Nikon, Japan).

### Long-term cell proliferation assays

Long-term cell proliferation assays were performed according to a previous study [25]. Long-term cell proliferation experiments were carried out in PC3 and DU145 cancer cell lines. First, 5.0 × 10^5^ cells were seeded into 10-cm^2^ dishes and incubated for 6 h to ensure that cells were completely adherent to the extracellular matrix. Second, PL at the indicated concentrations (i.e., 1.0, 2.0. or 4.0 μM) or DDP (4.0 μM) was added to the medium for another 72 h. Third, the cells were harvested, and the total number of cells was then counted. Finally, 5.0 × 10^5^ cells were reseeded into 10-cm^2^ dishes, and the above steps were repeated. The medium was changed every 3 days until confluence was reached. Quantification was performed as follows: 2^PDs^ = M/N, where PDs denote population doublings, M is the number of counted cells, and N is the number of implanted cells.

### Immunofluorescence (IF) assays

Immunofluorescence (IF) assays were performed as previously described [26]. Briefly, cells were fixed with 4% paraformaldehyde and permeabilized via 0.5% Triton X-100. Subsequently, cells were incubated with primary antibodies against focal adhesion kinase (FAK; CST) or 53BP1 (CST) and were then incubated with secondary antibodies (DyLight 488-conjugated anti-Rabbit). The cells were mounted with DAPI and/or phalloidin (CST). Fluorescent images were captured with a Nikon Ti microscope.

### Reactive oxygen species (ROS) assays

ROS was quantified using a ROS kit (Sigma) according to the manufacturer’s instructions. Cellular ROS levels were measured by flow cytometry. Briefly, 2 × 10^5^ cells were plated onto six-well cell culture plates and allowed to attach to the wells overnight. Thereafter, adhered cells were treated with PL (1.0, 2.0, or 4.0 μM) in the presence or absence of NAC (5 mM) pre-treatment for 2 h. After removal of the medium, the ROS indicator, DCFH-DA (10 μM), was added to fresh FBS-free medium and incubated for 30 min at 37 °C in the dark. Cells were then collected, and fluorescence was analyzed using a FACS Calibur flow cytometer (BD Biosciences, CA).

### DAPI staining

After long-term cell proliferation assays, PC3 and DU145 cells were re-seeded in six-well cell culture plates at a density of 5 × 10^5^ cells/well. After 6 h of incubation (during which cells adhered completely to the extracellular matrix), cells were fixed with 4% paraformaldehyde at room temperature for approximately 15 min, after which they were washed three times with PBS prior to DAPI staining. Cells were observed using a Nikon fluorescent microscope.

### Comet assays

DNA damage was evaluated by comet assays [27]. Cells were first mixed with 0.5% low-melting-temperature agarose before being transferred onto slides, which were coated with 1.5% normal agarose. Then, cells on these slides were lysed in 2.5-M NaCl, 10-mM Tris (pH 8.0), 100-mM EDTA, 0.5% Triton X-100, 1% N-lauroylsarcosine, and 3% DMSO. Electrophoresis was carried out in 300-mM sodium acetate, 100-mM Tris-HCl, and 1% DMSO. The slides were then mounted with PI solution (20 μg/ml) and visualized under a Nikon fluorescent microscope and analyzed by CASP.

### Western blotting

Total proteins were extracted and boiled for 10 min at 95 °C. Samples were separated via SDS-PAGE gels, transferred to a PVDF membrane, and then blocked with 5% nonfat milk in TBST for 2.5 h at room temperature. Blots were subsequently probed with relevant primary antibodies against FAK (CST), γ-H2AX (Millipore), cleaved PARP (CST), Bcl2 (Millipore), p53 (Santa), RPA (Santa), KU70 (Santa), XRCC4 (Santa) and β-actin (Proteintech) overnight at 4 °C. Finally, the blots were detected with HRP-conjugated secondary antibodies and visualized using a Westar Supernova kit (Cyanagen).

### Statistical analysis

GraphPad Prism 5 was used for statistical analysis. In histograms, all data were represented by mean ± SD of at least three replicates for each experiment. The statistical significance of the data was assessed by student’s two-tailed unpaired *t*-test or Two-way ANOVA with the *p*-values (*p < 0.05; **p < 0.01; ***p < 0.001).

## Results

### PL possesses stronger anticancer activity than that of taxol, cisplatin (DDP), doxorubicin (dox), or 5-fluorouracil (5-FU) in CRPC cells, with fewer side effects in normal cells

First, the effects of PL on cell viabilities of CRPC cells (PC3 and DU145), the normal prostatic stromal myofibroblast cell line (WPMY-1) and the normal hepatic cell line (LO2) were evaluated. Taxol, DDP, Dox, and 5-FU were selected as positive-control drugs in the present study, as they are the most widely used chemotherapeutic drugs against CRPC that are used clinically. The results showed that PL possessed stronger or comparable anticancer activity with fewer side effects in normal cells when compared with those of all other positive-control drugs that were tested (Table 1 and Supplementary Figure 1).

**Table 1:** *IC*_50_ (μM) values were determined via the MTT assay^a^

Cell lines / compound	*IC*_50_ (μM)			
	PC3	DU145	WPMY-1	LO2
PL	6.75 ± 0.75	8.42 ± 0.98	8.73 ± 0.32	68.62 ± 5.38
Taxol	7.88 ± 0.89	7.26 ± 0.69	4.37 ± 0.18	34.70 ± 1.03
DDP	27.81 ± 4.53	8.83 ± 1.25	7.43 ± 0.85	11.58 ± 0.35
Dox	33.19 ± 1.68	41.50 ± 1.15	2.14 ± 0.15	80.61 ± 3.89
5-FU	96.33 ± 1.86	25.75 ± 2.53	97.24 ± 2.03	94.97 ± 4.84

^a^*IC*_50_ values were drug concentrations necessary for 50% inhibition of cell viability. Data are average ± standard deviations of at least three independent experiments. The drug treatment period was 48 h

### PL inhibits CRPC cell migration by modulating the expression and distribution of FAK

**Fig. 1:**
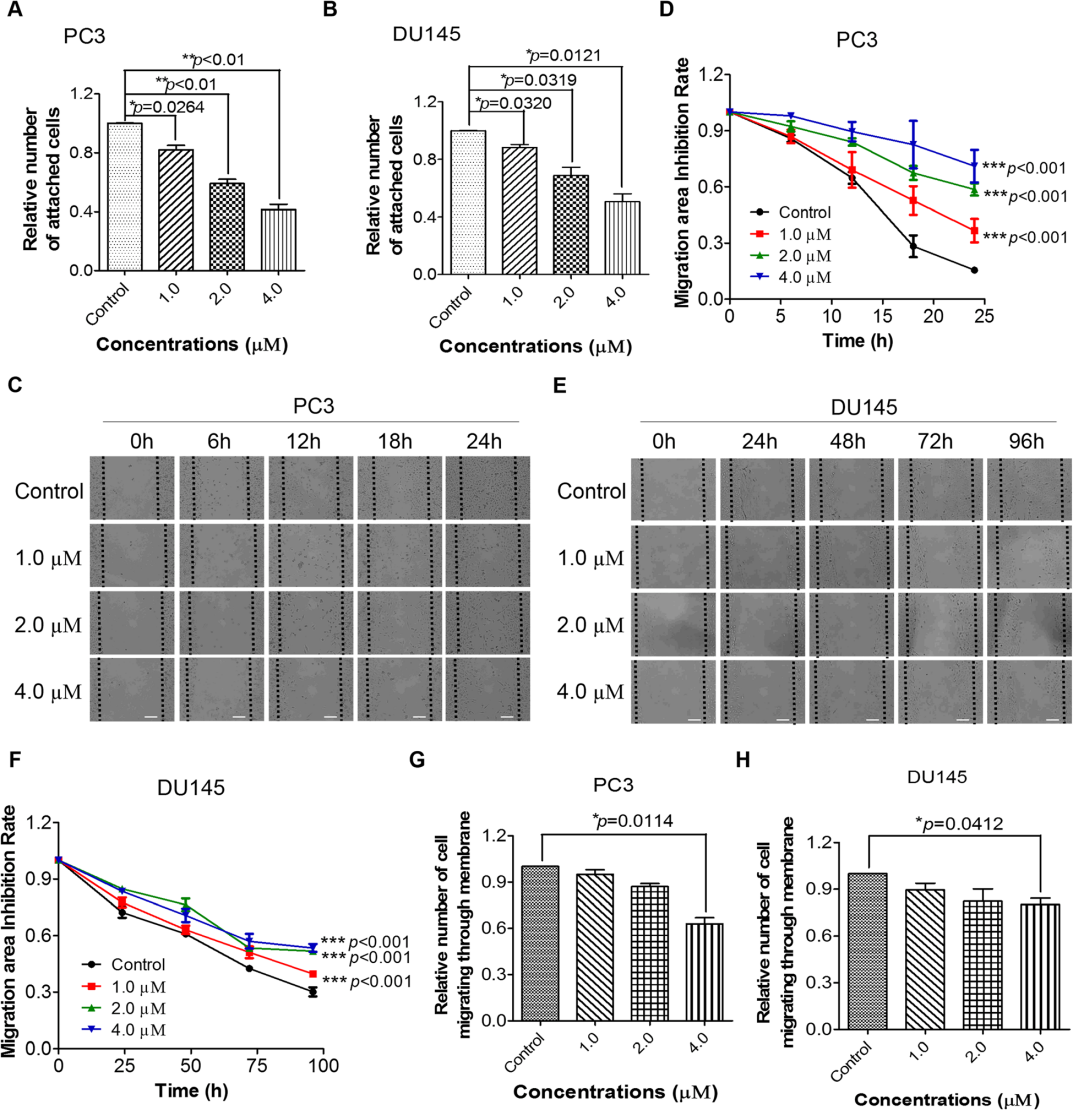
PL inhibits the migration of CRPC cells. **A** and **B**, PL decreases cell adhesion to the extracellular matrix in PC3 and DU145 cells. **C** and **E**, PL inhibits PC3 and DU145 cell migration in scratch-wound healing assays. The different time periods of PC3 and DU145 were adopted in scratch wound assay due to the difference migration speed between PC3 and DU145. Scale bar, 100 μm. **D** and **F**, Quantification of (**C**) and (E). **G** and **H**, PL inhibits PC3 and DU145 migration in transwell assays. Values represent the mean ± standard deviation (SD) of at least three independent experiments. Statistical significance was calculated using unpaired Student’s two-tailed *t* tests for **A**, **B**, **G**, **H**, and two-way analysis of variance (ANOVA) for **D** and **F** with the *p*-values (*p < 0.05, **p < 0.01, ***p < 0.001)

Cell adhesion assays were performed to determine the capability of cells to adhere to the extracellular matrix with or without PL treatment. As shown in Fig. 1A and B, PL treatment resulted in a significant concentration-dependent decrease in cell adhesion to the extracellular matrix at concentrations of 1.0, 2.0, and 4.0 μM.

Cell adhesion is known to be correlated with cell migration [28]. Therefore, scratch-wound healing assays were performed to determine the migration rate of PC3 and DU145 cells with or without PL treatment. The data indicated that PL significantly inhibited cancer cell migration (Fig. 1C and E). After a 24-h treatment, only 15% of the scratch area was uncovered by migrated PC3 cells in the control group, while 71% of the scratch area was uncovered following treatment with 4.0 μM of PL (Fig. 1D). Similarly, treatment with 4.0 μM induced a significantly lower migration rate in PL DU145 cells (Fig. 1F). To exclude the possibility of interference from cell death induced by PL, FACS assays and beta-galactosidase (SA-β gal) staining assays were used to evaluate apoptotic and senescent cells immediately after scratch-wound healing assays. The results showed that PL did not induce significant apoptosis or senescence during the cell migration assay (Supplementary Figure 2), indicating that PL-induced inhibition of cell migration was not induced by apoptosis/senescence.

In order to further determine the inhibitory effects of PL on PC3 and DU145 cell migration, transwell migration assays were carried out. The quantitative data suggested that PL-treated PC3 and DU145 cells possessed weaker transferability compared to that of untreated cells (Fig. 1G and H). These results were consistent with the above results from our scratch-wound healing assays (Fig. 1).

**Fig. 2:**
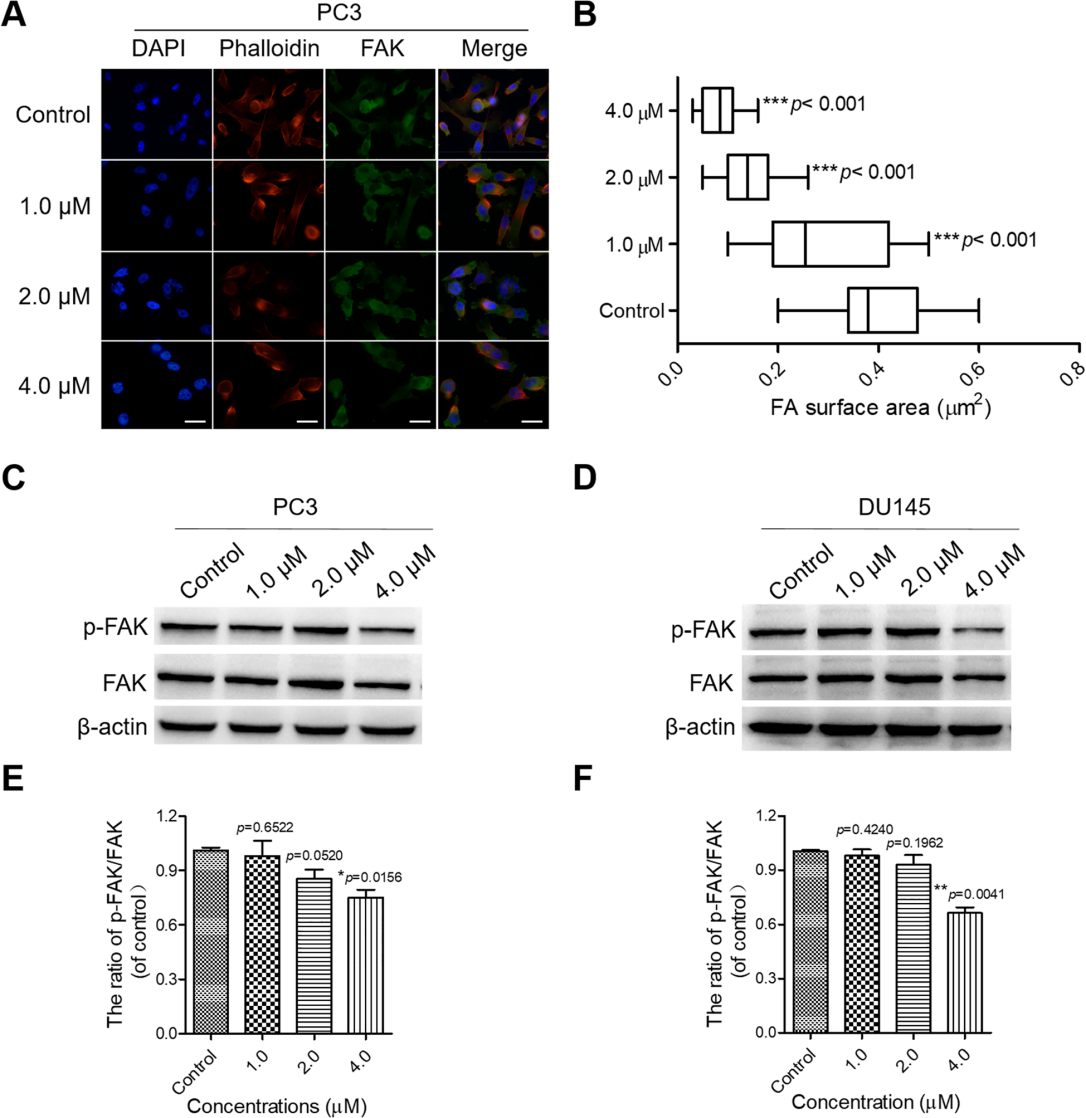
PL inhibits the expression and distribution of FAK in CRPC cells. **A** Representative images of FAK in PL-treated and untreated PC3 cells in IF assays. Cells were treated with the indicated concentration of PL (1.0, 2.0, or 4.0 μM) for 48 h. Cells treated with 0.01% DMSO were used as a control. Antibodies to FAK (blue) and phalloidin (red) were used to visualize FAK and F-actin, respectively. Scale bar, 10 μm. **B** The focal adhesion surface area was assessed through FAK and phalloidin staining in PL-treated and control PC3 cells. Cells were treated with the indicated concentration of PL (1.0, 2.0, or 4.0 μM) for 48 h. Values represent the mean ± SD of at least three independent experiments, and ≥ 500 cells were counted in each group. Statistical significance was calculated using unpaired student’s two-tailed *t* tests (*p < 0.05, **p < 0.01, ***p < 0.001). **C** and **D** Western blotting of FAK in control (0.01% DMSO) and PL-treated PC3 and DU145 cells. Cells were treated with the indicated concentration of PL (1.0, 2.0, or 4.0 μM) for 48 h and were used in Western-blot assays using an antibody against FAK. β-actin was adopted as a loading control. **E** and **F** Quantification of (**C**) and (**D**), respectively. The statistical significance was calculated using the unpaired student’s two-tailed *t*-test with the *p*-values (*p < 0.05, **p < 0.01, ***p < 0.001)

Since precisely controlled cell deformations are vital to cell migration [29], we speculated that PL might affect the expression and function of cytoskeletal proteins in PC3 and DU145 cells. F-actin is not only the most abundant cytoskeletal protein but also a crucial protein for cell stability, morphogenesis, and motility [29]. Meanwhile, FAK is a cytoplasmic kinase that is essential for cell migration and morphogenesis [30]. Both F-actin and FAK contribute to cell migration and adhesion; hence, we speculated that PL might inhibit CRPC cell migration and adhesion by affecting the expression and distribution of FAK and F-actin. Therefore, IF and Western-blot assays were performed using FAK and phalloidin antibodies. The IF results showed that weak fluorescence (FAK) was observed in PL-treated cells, whereas FAK was expressed at a much higher level in the control group (Fig. 2A and Supplementary Figure 3A). Moreover, PL-treated cells presented a decreased cell-spreading area in a concentration-dependent manner (Fig. 2B and Supplementary Figure 3B). In addition, Western blotting further showed that PL efficiently decreased the expression of FAK, especially p-FAK (Fig. 2C-F). Taken together, these findings support the conclusion that PL inhibited the migration of CRPC cells via suppressing the expression and distribution of FAK at the edge of the cells.

### PL effectively inhibits proliferation of CRPC cells and induces cell death by increasing ROS

**Fig. 3:**
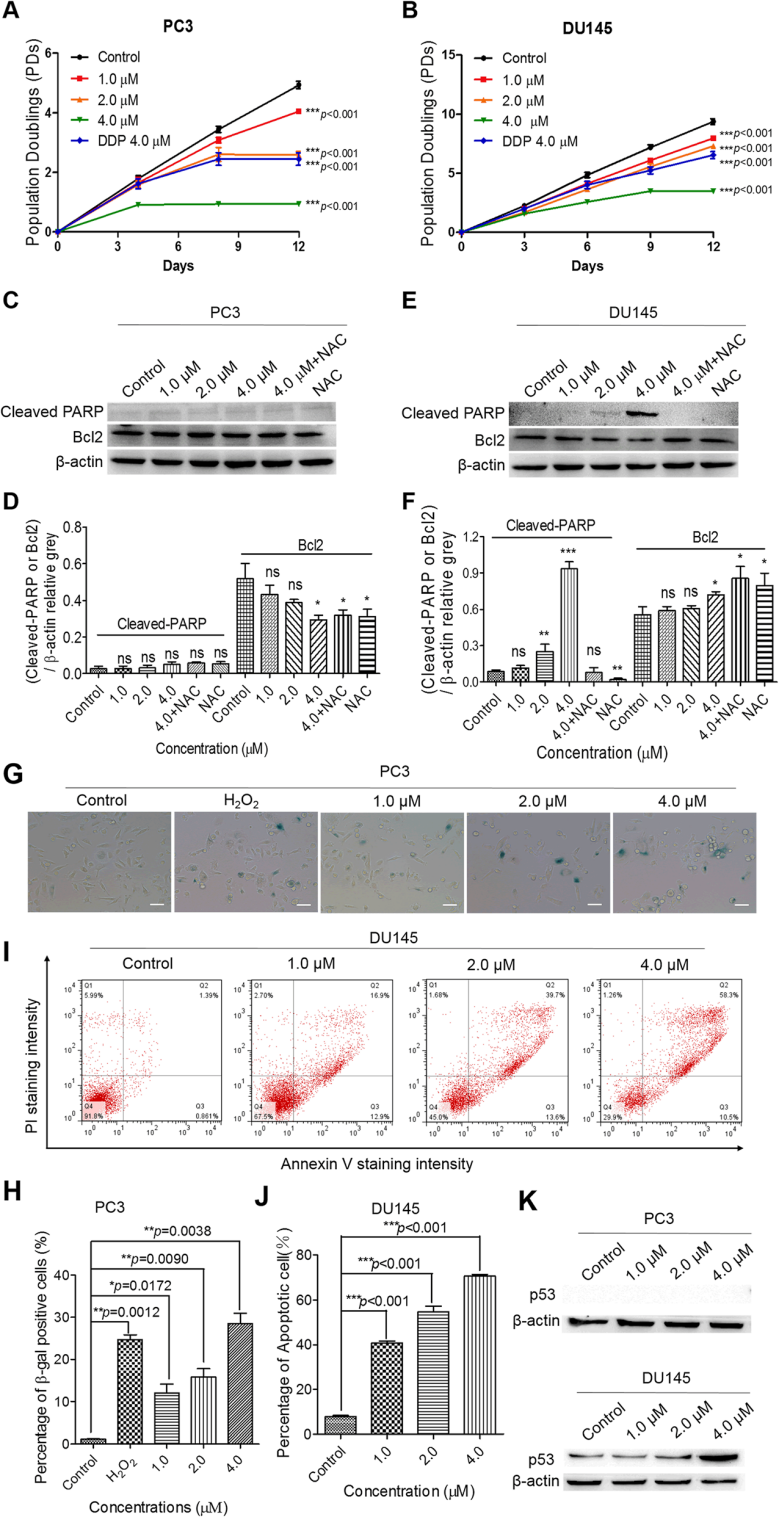
PL inhibits the proliferation of CRPC cells and induces cell death. Proliferation curves of PL-treated PC3 (**A**) and DU145 cells (**B**). Statistical significance was calculated using two-way ANOVA (*p < 0.05, **p < 0.01, ***p < 0.001). Western blotting of cleaved PARP and Bcl2 in control (0.01% DMSO) and PL-treated PC3 (**C**) and DU145 (**E**) cells. Cells were treated with the indicated concentration of PL (1.0, 2.0, or 4.0 μM) for 48 h and were subjected to Western blotting using an antibody against cleaved PARP or Bcl2. β-actin was used as a loading control. Cells were pretreated with 5 mM of NAC for 2 h before treatment with PL (4.0 μM). **D** and **F**, Quantification of C and E, respectively. Values represent the mean ± SD of at least three independent experiments. β-galactosidase staining assays were carried out to assess the senescence of PC3 (**G**) cells after PL treatment for 12 days. Scale bar, 100 μm. FACS analysis was performed to detect apoptotic DU145 (**I**) cells treated with the indicated concentration of PL (1.0, 2.0, or 4.0 μM) over 12 days. **H** and **J** represent quantification of G and I, respectively. **K** The expression of p53 was detected by WB assays at indicated concentration in PL-treated PC3 and DU145 cells. Statistical significance was calculated using unpaired student’s two-tailed *t* tests (*p < 0.05, **p < 0.01, ***p < 0.001)

High levels of ROS, which are correlated to cell fate, were found to induce both cell-cycle arrest and cell death in PL-treated PC3 and DU145 cells (Supplementary Figure 4). First, the effect of PL on cell proliferation was determined by long-term cell proliferation assays. The results indicated that the proliferation of PC3 and DU145 cells was significantly inhibited by PL in a concentration-dependent manner (Fig. 3A and B). Since the induction of apoptosis is associated with modulation of PARP [31] and Bcl-2 [32], we also visualized cleaved PARP and Bcl2 content via Western blotting. The results indicated that PL treatment had no noticeable effect on the expression of cleaved PARP but slightly down-regulated the expression of Bcl2, an important anti-apoptotic associated protein, in PC3 cells (Fig. 3C and D). Conversely, PL significantly increased the content of cleaved PARP protein at 4.0 μM but had little effect on Bcl2 in DU145 cells (Fig. 3E and F). As expected, elevated cleaved PARP protein expression was absent after pretreatment with the ROS scavenger, N-acetyl cysteine (NAC) (Fig. 3C and E). Taken together, these data indicate that PL treatment may result in different cell fates in PC3 and DU145 and that these effects are mediated by ROS, especially in PL-treated DU145 cells.

The status of the tumor-suppressor gene, p53, is null in PC3 cells but mutated in DU145 cells, and contributes to differential cell fates [33–35]. Therefore, both annexin V/PI apoptotic assays and SA-β gal staining assays (Fig. 3G-J and Supplementary Figure 5) were carried out to detect the cell fates of PC3 and DU145 cells. H_2_O_2_-treated cells were used as a positive control (Fig. 3G and Supplementary Figure 5C). The flow cytometry results showed that immediately after long-term cell proliferation assays (PL treatment for 12 days), the apoptotic ratio was not changed in PL-treated PC3 cells (Supplementary Figure 5A and B), whereas PL significantly induced cell senescence (Fig. 3G and H). Conversely, PL treatment induced apoptosis in DU145 cells with concomitantly increased expression of p53 (Fig. 3I-K), while senescent cells were undetectable (Supplementary Figure 5C and D). Furthermore, we examined nuclear morphology in PL-treated or untreated CRPC cells immediately after long-term proliferation assays. The results showed that more than 70% of PL-treated DU145 cells had apoptotic nuclei (73.2 ± 2.1%), including condensed nuclei (Supplementary Figure 6). In contrast, PL-treated PC3 cells and controls appeared to exhibit intact oval-shaped morphologies, and their nuclei did not appear to be as apoptotic.

### PL treatment induces persistent DNA damage

**Fig. 4:**
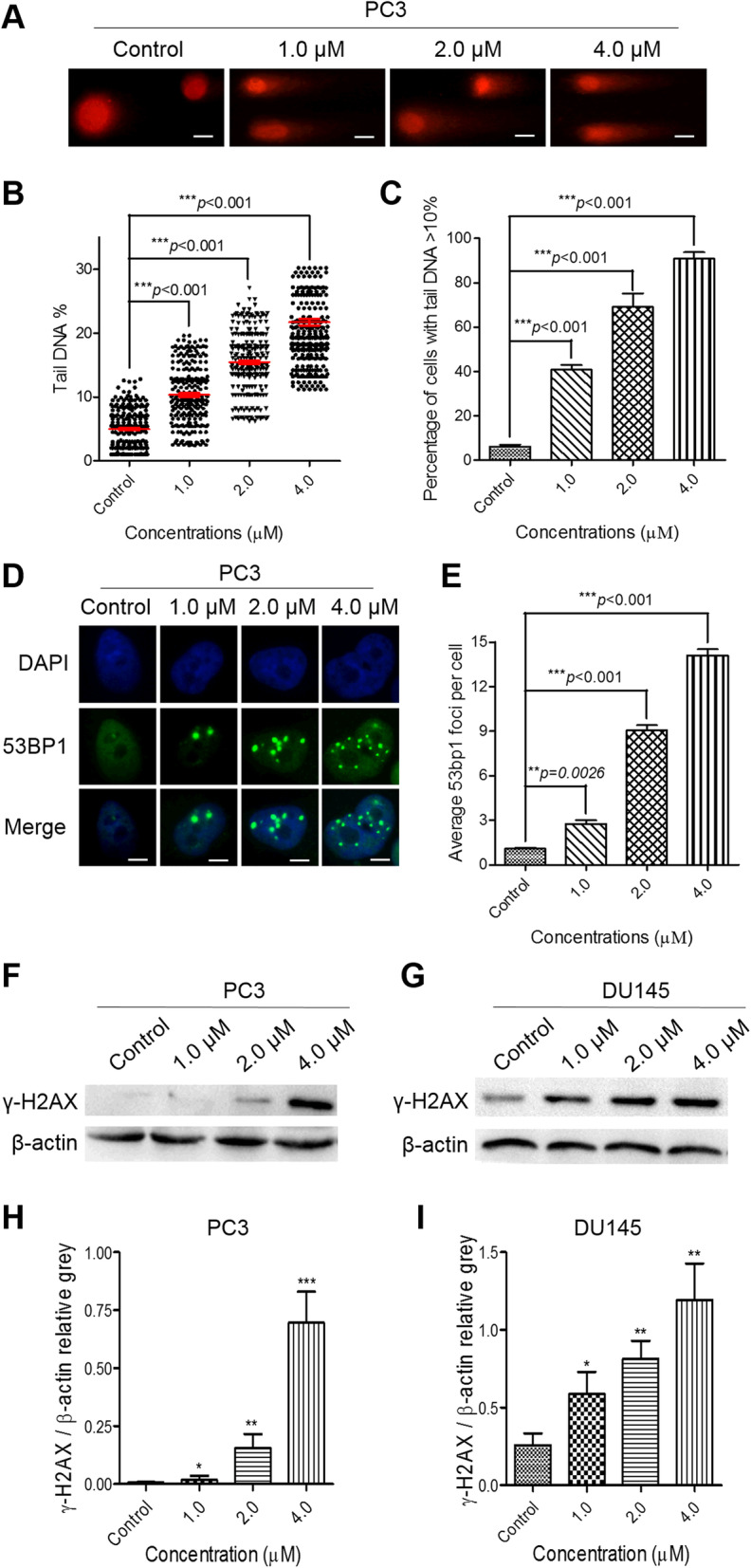
PL treatment triggers strong DNA damage and provokes an intense DDR in CRPC PC3 cells. **A** The data from comet assays show that PL treatment triggered strong DNA damage in a concentration-dependent manner. Scale bar, 100 μm. **B** and **C**, The percentages of tail DNA in PL-treated PC3 cells (**B**) and the number of PL-treated PC3 cells containing more than 10% tail DNA (**C**) were measured. Cells were treated with the indicated concentration of PL (1.0, 2.0, or 4.0 μM) for 48 h, and ≥ 500 cells were examined in each group. **D** The IF data show that PL treatment provoked an intense DDR in a concentration-dependent manner. Scale bar, 10 μm. **E** Quantification of (**D**). Cells were treated with the indicated concentration of PL (1.0, 2.0, or 4.0 μM) for 48 h, and ≥ 200 cells were examined in each group. **F** and **G** Western blotting of γ-H2AX in control (0.01% DMSO) and PL-treated PC3 (**F**) and DU145 (**G**) cells. Cells were treated with the indicated concentration of PL (1.0, 2.0, or 4.0 μM) for 48 h and were subjected to Western blotting using an antibody against γ-H2AX. β-actin was used as a loading control. **H** and **I**, Quantification of (**F**) and (**G**), respectively. Values represent the mean ± SD of at least three independent experiments. The statistical significance was calculated using the unpaired student’s two-tailed *t*-test with the *p*-values (*p < 0.05, **p < 0.01, ***p < 0.001)

FAK and ROS can affect DNA damage as well as the DNA damage response (DDR) [36, 37], and unbalanced DNA damage and repair will result in apoptosis and/or senescence [33]. Therefore, comet assays and IF assays were carried out to examine the effects of PL on DNA damage and the DDR in CRPC cells. As expected, PL caused increased DNA damage in PC3 cells, resulting in DNA fragments that formed a tail shape during electrophoresis (Fig. 4A-C). These results were consistent with the data from our IF assays, which showed that the number of 53BP1 foci in PL-treated PC3 cells was significantly increased in a concentration-dependent manner (Fig. 4D and E). In addition, similar results were obtained in PL-treated DU145 cells (Supplementary Figure 7). The increased expression of γ-H2AX, a marker of DNA double strand breaks (DSB), in PL-treated CRPC cells (Fig. 4F-I) further supported the conclusion that PL triggered substantial DNA damage and the DDR in CRPC cells. Moreover, we found that PL treatment resulted in no change in amount of replication protein A (RPA), which plays a role in DNA strand invasion during homologous recombination (HR) [38]. In contrast, PL treatment suppressed the expression of KU70 and XRCC4, the marker of non-homologous end joining (NHEJ) [39], in a concentration-dependent manner (Supplementary Figure 8A and B). Additionally, we also tested DNA damage in PL-treated, positive-control-treated, or untreated human normal LO2 cells using an antibody against 53BP1; subsequently, the number of foci per nucleus was then calculated. Our data showed that compared to those following taxol, DDP, Dox, or 5-FU treatments, less 53BP1 foci were detected in PL-treated LO2 cells, indicating that PL induced less DNA damage in human normal LO2 cells (Supplementary Figure 9).

**Fig. 5:**
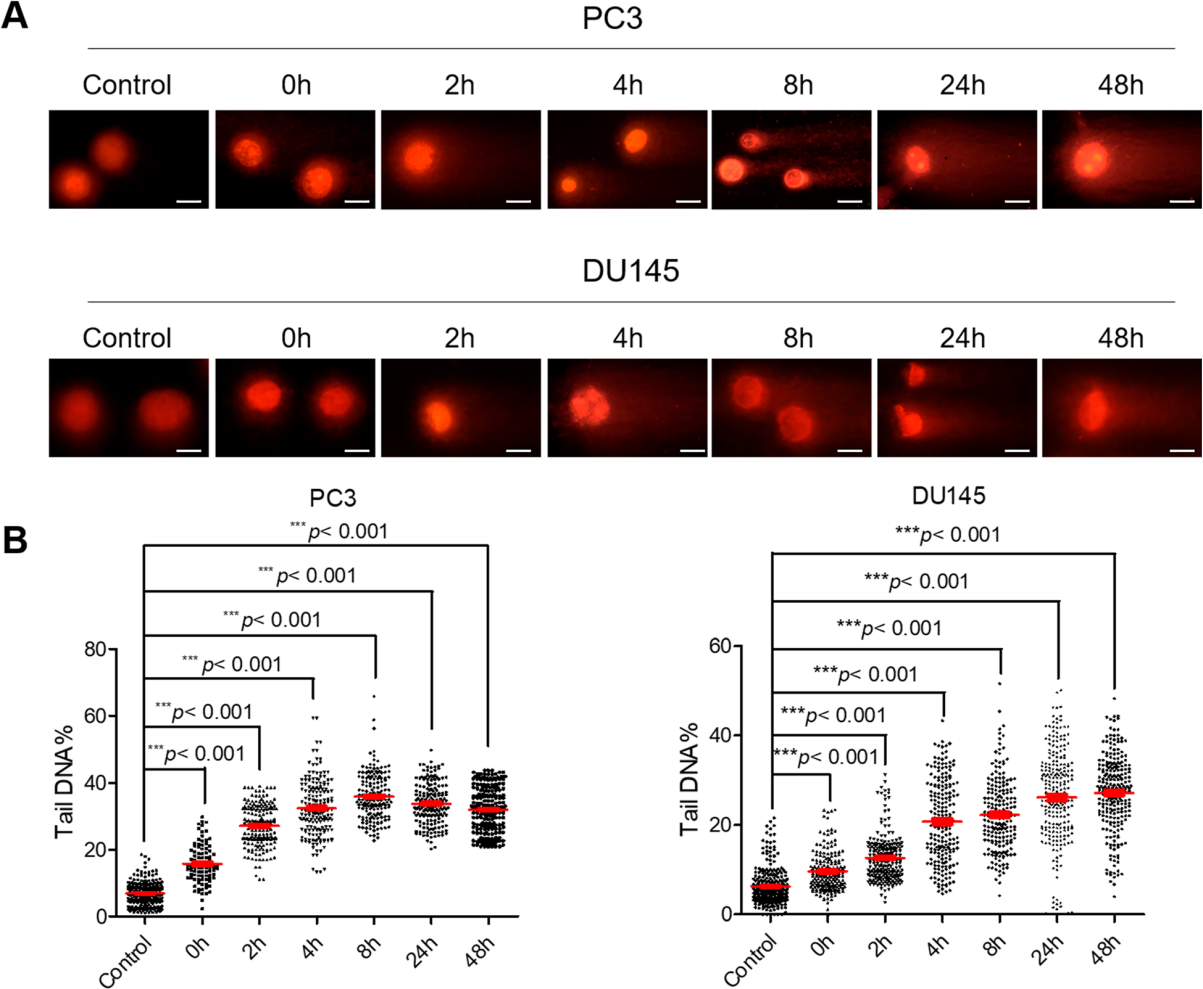
PL-induced DNA damage is not repaired in CRPC PC3 and DU145 cells. **A** Representative images of DNA damage via comet assays. PC3 or DU145 cells were treated with PL (10 μM) for 3 h and were then collected at the indicated times (0, 2, 4, 8, 24, or 48 h). Scale bar, 100 μm. **B** The percentages of tail DNA in PL-treated PC3 or DU145 cells were measured. Values represent the mean ± SD of at least three independent experiments, and ≥ 500 cells were counted in each group. Statistical significance was calculated using unpaired Student’s two-tailed *t* tests (*p < 0.05, **p < 0.01, ***p < 0.001)

To explore the consequences of DNA damage, both PC3 and DU145 cells were treated with PL at a dose of 10 μM for 3 h. Cells under this condition did not exhibit any senescence (Supplementary Figure 10A and B), apoptosis (Supplementary Figure 10C and D), or cell-cycle arrest (Supplementary Figure 10E-H). After PL treatment, the media was immediately replaced with PL-free medium, and cells were fixed at the indicated times (0, 2, 4, 8, 24, or 48 h). The fixed cells were then used in comet assays and IF assays. The data from comet assays showed that PL treatment triggered numerous DNA lesions in PC3 and DU145 cells, resulting in DNA lesions that left the nucleus and formed tail shapes during the comet assay (Fig. 5A). It is noteworthy that these tail shapes were still present within 48 h after PL treatment (Fig. 5B), suggesting suppressed repair of DNA damage in PL-treated CRPC cells and/or PL-induced persistent DNA damage in CRPC cells.

**Fig. 6:**
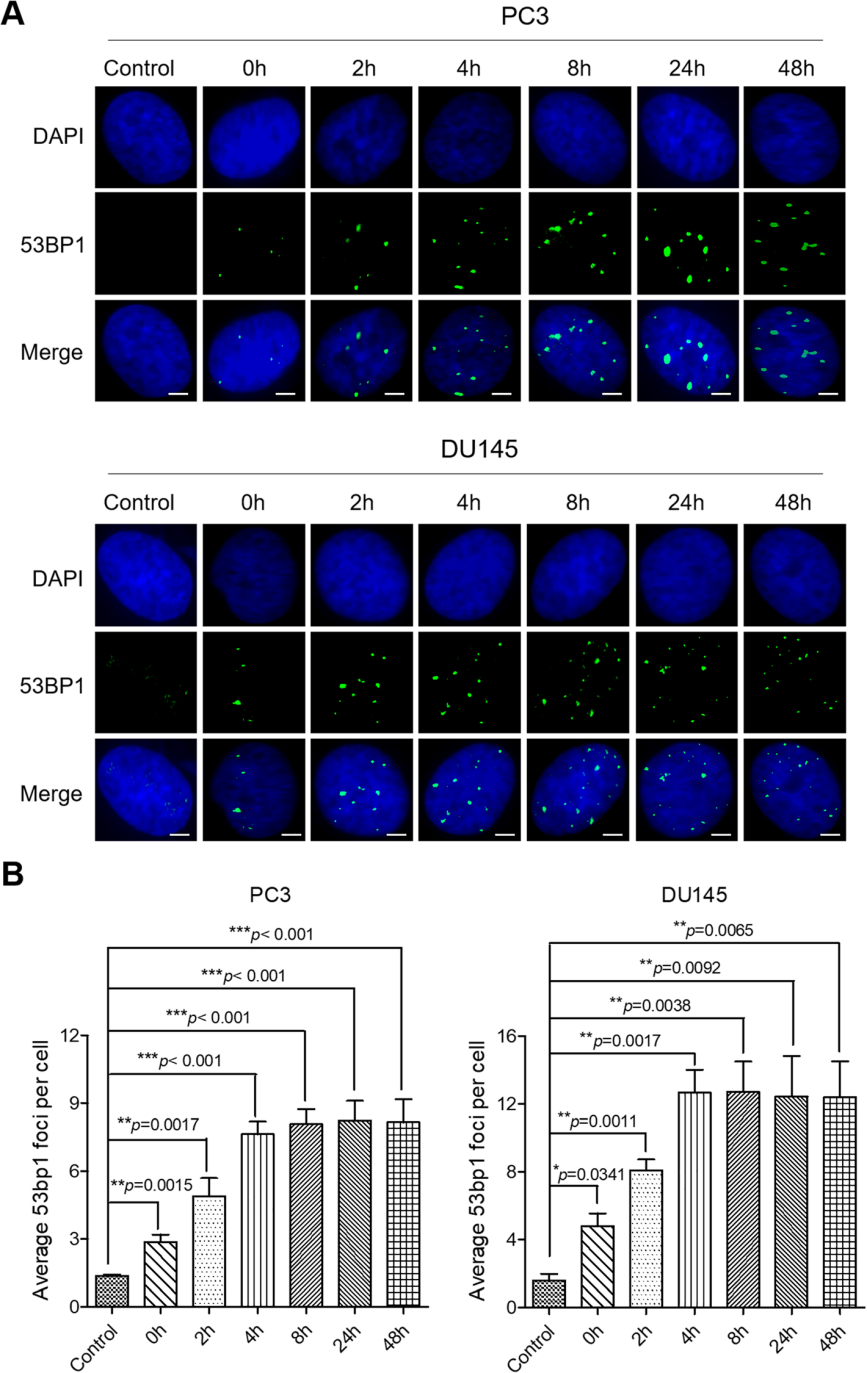
DDR machinery is impaired in PL-treated CRPC cells. **A** Cells were exposed to PL (10 μM) for 3 h and then replaced with PL-free medium for 0, 2, 4, 8, 24, and 48 h. Untreated cells served as a control. 53BP1 foci were calculated by IF using an 53BP1 antibody. Cell nuclei were stained using DAPI. Scale bar, 10 μm. **B** Quantification of (**A**). The average number of 53BP1 foci per cell was determined and ≥ 200 cells were counted each group. Values are average ± SD of at least three independent experiments. The statistical significance was calculated using the unpaired student’s two-tailed *t*-test with the *p*-values (*p < 0.05, **p < 0.01, ***p < 0.001)

We also used IF assays to monitor DNA damage repair in PL-treated CRPC cells (Fig. 6A). The number of 53BP1 foci achieved the highest value at 4 h after PL treatment in both PC3 and DU145 cells (8 and 13 foci per nucleus, respectively) (Fig. 6B). However, these values were still maintained within 48 h after PL-treatment (Fig. 6A and B). Next, we performed western blots to examine the abundance of HR-related protein RPA and NHEJ-related protein XRCC4 in 10 μM PL treated-PC3 and DU145 cells at different time (0 h, 4 h, 8 h, 24 h and 48 h). The results showed that 10 μM PL treatment obviously reduced expression of RPA and XRCC4, and increased expression of p53 (Supplementary Figure 11A and B). These results revealed that DNA damage induced by PL may not be repaired in CRPC cells. Consequently, Annexin V/PI apoptotic assays indicated that 10 μM PL significantly induces DU145 cells apoptosis in time-dependent manner, but slightly induces apoptosis in PC3 cells, which may attribute to the expression of p53 (Supplementary Figure 11C-F). Taken together, these data support the conclusion that PL treatment induced intense DNA damage in CRPC cells.

## Discussion

Androgen deprivation (ADT) is a major strategy for mitigating advanced prostate cancer; however, the cure success rate of ADT is low, and most patients eventually develop CRPC [6]. Moreover, 90% of CRPC patients develop metastases with a high mortality rate, for which chemotherapy is the preferred clinical treatment [1]. Chemotherapy achieves anticancer effects via causing substantial DNA damage [9]. However, chemotherapy often fails after long-time treatment due to enhanced capacities of DNA repair in cancer cells [9]. Additionally, long-time chemotherapy often leads to serious toxic side effects. Thus, there has been an urgent need to identify a chemotherapeutic drug that possesses preferential effects against cancer cells without causing serious adverse side effects in normal cells. Our present results indicate that PL exhibited a broad spectrum of antitumor activities in CRPC cells, independent of p53, and induced lower cytotoxicity than that of cisplatin or taxol in normal cells, as well in WPMY-1 and LO2 cells (Table 1 and Supplementary Figure 9).

Considering that migration is the leading cause of cancer-related deaths [5, 28], a drug capable of effectively inhibiting the migration of cancer cells has potential therapeutic value. In our present study, we demonstrated that PL-treated CRPC cells exhibited decreased adhesion to the extracellular matrix (Fig. 1A and B). Accordingly, a decreased migration speed (Fig. 1C-F) and an attenuated ability to traverse a membrane from the serum-free to serum side (10% FBS) in the transwell assay (Fig. 1G and H) were observed in PL-treated CRPC cells. FAK, a non-receptor protein tyrosine kinase, is overexpressed and activated in a majority of cancers, including prostate cancer. FAK localizes to focal adhesions and it is activated by extracellular signals including integrin-mediated adhesion, which further leads phosphorylation of Tyr397 and activate downstream signaling pathways, contributes to the metastasis and invasion of prostate cancer cells [40]. Our study showed that PL inhibited the expression and distribution of FAK at the leading edge of cells as well as inhibited phosphorylation of Tyr397, and PL-treated cells presented a decreased cell-spreading area in a concentration-dependent manner (Fig. 2 and Supplementary Figure 3). Given the importance of F-actin and FAK in cell adhesion and migration, we propose that PL inhibited the adhesion and migration of CRPC cells by suppressing the expression and distribution of FAK in CRPC cells. Therefore, FAK is a potential effective target to inhibit migration of prostate cancer.

Previous studies have demonstrated that PL exhibited potent anticancer effects by activating ROS [13, 41] and high ROS levels induce substantial DNA damage [37]. Since the integrity of DNA is crucial to cell viability and metastasis of cancer cells, sufficient DNA damage can cause cell-cycle arrest and cell death [7]. The results of our present study indicated that PL suppressed CRPC cell proliferation and induced cell death (Fig. 3) at low concentrations (i.e., 1.0, 2.0, and 4.0 μM) by provoking intense DNA damage and inducing a strong DDR and inhibiting the DNA repair process (Fig. 4, Supplementary Figure 7 and Supplementary Figure 8). Furthermore, we found that the different cell fates of PL-treated PC3 (senescence) and DU145 (apoptosis) cells may be the result of multiple factors, including a differential p53 status between these cell types (Fig. 3K), as well as differential changes in PL-induced protein expression and apoptosis (Fig. 3C and D). Taken together, these data suggest that PL might achieve anticancer effects through ROS-mediated DNA damage and inhibition of repair pathways in a p53-independent manner. Therefore, the PL-induced fate of these DNA lesions in CRPC cells is worthy of further investigation.

Cancer cells have a high tolerance to genotoxicity via DNA repair and/or reversal of epigenetic defects [7]. In addition, an imbalance in redox equilibrium may cause serious DNA damage, and FAK inhibition can also result in persistent DNA damage [36, 37]. As expected, our present results demonstrated that PL induced substantial DNA damage in CRPC cells (Figs. 5 and 6), which may have been attributed to a substantial imbalance in redox equilibrium (Supplementary Figure 4) and FAK inhibition (Fig. 2 and Supplementary Figure 3). In addition, DNA damage repair approach including HR and NHEJ for DSB were impeded in PL treated PC3 and DU145 cells (Supplementary Figure 8, Supplementary Figure 11A and B). As a result, substantial DNA damage and DDR accumulation-initiated inhibition of cell migration (Fig. 1) and a cell death response (Fig. 3).

## Conclusions

In conclusion, our present results found that PL efficiently inhibited the migration and proliferation of CRPC cells and induced cell death in a concentration-dependent manner. Moreover, the results indicated that PL treatment would regulate the expression and distribution of FAK and the intracellular ROS levels. Further mechanism studies showed that PL achieved both anti-proliferation and anti-migration activities by triggering intense DNA damage in CRPC cells.

## Supplementary Information

**Additional file 1: Supplementary** **Figure 1.** *IC*_50_ (μM) values were determined via the MTT assay. *IC*_50_ values were drug concentrations necessary for 50% inhibition of cell viability. Data are average ± standard deviations of at least three independent experiments. The drug treatment period was 48 h. **Supplementary Figure 2.** Piperlongumine (PL) treatment did not induce significant cell apoptosis or senescence after the cell migration assay. FACS analysis was performed to detect the apoptotic PC3 **(A)** and DU145 **(C)** cells after the scratch-wound assay with the indicated concentration of PL (1.0 μM, 2.0 μM or 4.0 μM). β-galactosidase staining assay was carried out to assess the senescence of PC3 **(E)** and DU145 **(G)** cells after the scratch-wound assay with the indicated concentration of PL (1.0 μM, 2.0 μM or 4.0 μM). Scale bar, 100 μm. **(B)**, **(D)**, **(F)** and **(H)** were quantification of A, C, E and G, respectively. Values are average ± SD of at least three independent experiments. The statistical significance was calculated using the unpaired student’s two-tailed t-test with the *p*-values (*p < 0.05, **p < 0.01, ***p < 0.001). **Supplementary Figure 3.** Piperlongumine (PL) inhibits the expression and distribution of focal adhesion kinase (FAK) in castration-resistant prostate cancer (CRPC) DU145 cells. **(A)**, Representative images of focal adhesion kinase (FAK) in PL-treated and control DU145 cells in immunofluorescence assays. Cells were treated with the indicated concentration of PL for 48 h. Cells treated with 0.01% DMSO were used as a control (Control). Antibody to FAK (blue) and phalloidin (red) were used to visualize FAK and F-actin, respectively. Scale bar, 10 μm. **(B)**, Focal adhesion surface area assessed through FAK and phalloidin staining in PL-treated and control DU145 cells. Cells were treated with indicated concentration of PL (1.0 μM, 2.0 μM or 4.0 μM) for 48 h. Values are average ± SD of three independent experiments and ≥ 500 cells were examined in each group. The unpaired student’s two-tailed t-test was used to determine the statistical significance (*p < 0.05, **p < 0.01). **Supplementary Figure 4.** Piperlongumine (PL) generates reactive oxygen species (ROS) in castration-resistant prostate cancer (CRPC) PC3 and DU145 cells. **(A)**, Intracellular ROS generation in PC3 cells exposed to PL. Cells were treated with 1.0, 2.0 or 4.0 μM PL for 48 h and then stained with ROS probe DCFH-DA. NAC pretreatment was carried out at 5 mM for 1 h. Representative histogram is shown. **(B)**, Quantification of ROS levels in PC3 cells as determined by DCFH-DA probe. **(C)**, The same as A, except DU145 cells were used. **(D)**, Quantification of ROS levels in DU145 cells as determined by DCFH-DA probe. Values are average ± SD of three independent experiments. The unpaired student’s two-tailed t-test was used to determine the statistical significance (*p < 0.05, **p < 0.01, ***p < 0.001). **Supplementary Figure 5.** PL treatment induces cell death in CRPC cells. FACS analysis was performed to detect apoptotic PC3 (A) cells treated with the indicated concentration of PL (1.0, 2.0, or 4.0 μM) over 12 days. β-galactosidase staining assays were carried out to assess the senescence of DU145 (C) cells after PL treatment for 12 days. (B) and (D) represent quantification of A and C, respectively. Values represent the mean ± SD of at least three independent experiments. The statistical significance was calculated using the unpaired student’s two-tailed t-test with the *p*-values (*p < 0.05, **p < 0.01, ***p < 0.001). **Supplementary Figure 6.** DAPI stained PC3 and DU145 cells after treatment of PL at indicated concentrations (1.0, 2.0, or 4.0 μM) immediately after long-term cell proliferation of incubation. Scale bar, 100 μm. **Supplementary Figure 7.** Piperlongumine (PL) treatment triggers strong DNA damage and provokes intense DNA damage response in prostate cancer (CRPC) DU145 cells. (**A**) The data from comet assay showed that PL treatment triggers strong DNA damage in a concentration-dependent manner. Scale bar, 100 μm. (**B**) and (**C**), the percentages of DNA in the tail for PL treated DU145 cells (**B**) and the percentage of PL treated DU145 with over 10% tail DNA (**C**) were measured. (**D**) The data from immunofluorescence showed that PL treatment provokes intense DNA damage response in a concentration-dependent manner. Scale bar, 10 μm. (**E**) Quantification of (**D**). Cells were treated with indicated concentration of PL (1.0, 2.0, or 4.0 μM) for 48 h and ≥ 200 cells were examined in each group. Values are average ± SD of three independent experiments. The unpaired student’s two-tailed t-test was used to determine the statistical significance (*p < 0.05, **p < 0.01, ***p < 0.001). **Supplementary Figure 8.** Western blot assay for evaluating the expression of RPA, XRCC4 and KU70 in indicated concentration PL treated PC3 and DU145 cells. (**A**). The expression of RPA, XRCC4 and KU70 in PC3 cells. (**B**). The expression of RPA, XRCC4 and KU70 in DU145 cells. **Supplementary Figure 9.** Immunofluorescence (IF) assay for evaluating the DNA damage. (**A**) LO2 cells were treated with 0.01% DMSO (Control), piperlongumine (PL, 4.0 μM), taxol (4.0 μM), cisplatin (DDP, 4.0 μM), doxorubicin (Dox, 4.0 μM), 5-Fluorouracil (5-FU, 4.0 μM) for 48 h. DAPI and 53BP1 was the nucleus dye (blue) and DNA damage marker (green), respectively. Scale bar, 10 μm. (**B**) Quantification of A. The results show the percentage of 53BP1 foci per cell among 200 untreated and treated cells, respectively. The Bar chart of all data represents mean ± SD of three independent experiments, *p < 0.05, **p < 0.01 and ***p < 0.001. **Supplementary Figure 10.** Piperlongumine (PL) treatment (10μM, 3 h) did not induce the senescence, the apoptosis and/or cell arrest of castration-resistant prostate cancer (CRPC) PC3 and DU145 cells. (**A**) Representative results of β-gal staining assay of PL treated PC3 cells. Scale bar, 100 μm. (**B**) Quantitation of the percentage of senescent (β-gal positive) cells in A. (**C**) The percentage of apoptotic cells in PL treated DU145 cells were analyzed by flow cytometry. **(D)** Quantitation of the percentage of apoptotic (Annexin V positive) cells in C. (**E**), Cell cycle arrest of PL treated PC3 cells were detected by FACS. **(F)** Quantitation of E. (**G**) As in E, except DU145 cells were used. **(H)** Quantitation of G. Control indicates cells without PL treatment and values are average ± SD of three independent experiments. **Supplementary Figure 11.** 10 μM PL treatment at indicated time suppressed the process of DNA damage repair in PC3 and DU145 cells and induced apoptosis in DU145 cells. (**A**), (**B**) The expression of p53, RPA and XRCC4 in PC3 and DU145 cells, respectively. (**C**)**, (D)** The percentage of apoptotic cells in PL treated DU145 and PC3 cells were analyzed by flow cytometry, respectively. (**E**)**, (F)** Quantitation of (C) and (D), respectively.

**Additional file 2.** Original blot images.

## Data Availability

The datasets used and analyzed during the current study available from the corresponding author on reasonable request.

## Abbreviations

CRPC: Castration-resistant prostate cancer
ADT: Androgen-deprivation therapy
DDP: Cisplatin
DDR: DNA damage response
Dox: Doxorubicin
5-FU: 5-Fluorouracil
MTT: 3-(4,5-dimethyl-2-thiazolyl)-2,5-diphenyl-2-H-tetrazolium bromide
IF: Immunofluorescence
PARP: Poly (ADP-ribose) polymerase
PL: Piperlongumine
PSA: Prostate-specific antigen
ROS: Reactive oxygen species
FAK: Focal adhesion kinase
SA-β gal: Senescence-associated beta-galactosidase activity
