# Supplementary material for: Piperlongumine inhibits migration and proliferation of castration-resistant prostate cancer cells via triggering persistent DNA damage
Source: BMC Complement Med Ther. 2021 Jul 6;21:195. doi: 10.1186/s12906-021-03369-0 (PMC8261967; doi:10.1186/s12906-021-03369-0)
Supplement: Supplementary file 1 — Additional file 1: Supplementary Figure 1. IC50 (μM) values were determined via the MTT assay. IC50 values were drug concentrations necessary for 50% inhibition of cell viability. Data are average ± standard deviations of at least three independent experiments. The drug treatment period was 48 h. Supplementary Figure 2. Piperlongumine (PL) treatment did not induce significant cell apoptosis or senescence after the cell migration assay. FACS analysis was performed to detect the apoptotic PC3 (A) and DU145 (C) cells after the scratch-wound assay with the indicated concentration of PL (1.0 μM, 2.0 μM or 4.0 μM). β-galactosidase staining assay was carried out to assess the senescence of PC3 (E) and DU145 (G) cells after the scratch-wound assay with the indicated concentration of PL (1.0 μM, 2.0 μM or 4.0 μM). Scale bar, 100 μm. (B), (D), (F) and (H) were quantification of A, C, E and G, respectively. Values are average ± SD of at least three independent experiments. The statistical significance was calculated using the unpaired student’s two-tailed t-test with the p-values (*p < 0.05, **p < 0.01, ***p < 0.001). Supplementary Figure 3. Piperlongumine (PL) inhibits the expression and distribution of focal adhesion kinase (FAK) in castration-resistant prostate cancer (CRPC) DU145 cells. (A), Representative images of focal adhesion kinase (FAK) in PL-treated and control DU145 cells in immunofluorescence assays. Cells were treated with the indicated concentration of PL for 48 h. Cells treated with 0.01% DMSO were used as a control (Control). Antibody to FAK (blue) and phalloidin (red) were used to visualize FAK and F-actin, respectively. Scale bar, 10 μm. (B), Focal adhesion surface area assessed through FAK and phalloidin staining in PL-treated and control DU145 cells. Cells were treated with indicated concentration of PL (1.0 μM, 2.0 μM or 4.0 μM) for 48 h. Values are average ± SD of three independent experiments and ≥ 500 cells were examined in each group. The unpaired st [file 12906_2021_3369_MOESM1_ESM.docx]

Piperlongumine inhibits migration and proliferation of castration-resistant prostate cancer cells via triggering persistent DNA damage

Ding-fang Zhang^1#^, Zhi-chun Yang^1,2#^, Jian-qiang Chen^1#^, Xiang-xiang Jin^1^, Yin-da Qiu^1^, Xiao-jing Chen^1^, Hong-yi Shi^1^, Zhi-guo Liu^1^, Minshan Wang^3, 4^, Guang Liang^1^**, Xiao-hui Zheng^1^*

^1^Chemical Biology Research Center, School of Pharmaceutical Sciences, Wenzhou Medical University, Wenzhou 325035, Zhejiang, People’s Republic of China

^2^The Fifth Affiliated Hospital of Wenzhou Medical University, Affiliated Lishui Hospital of Zhejiang University, The Central Hospital of Zhejiang Lishui, Lishui 323000, Zhejiang, People’s Republic of China.

^3^The Affiliated Xiangshan Hospital, Wenzhou Medical University, Ningbo 315000, Zhejiang, China

^4^Hospital of Chinese Medicine of Haishu District, Ningbo 315000, Zhejiang, China

**^#^** These authors contributed equally to this work.

***** *Correspondence***:**Prof. Guang Liang and/or Associate prof. Xiao-hui Zheng
wzmcliangguang@163.com and/or zhengxh@wmu.edu.cn.

**Supplementary** **Figure 1.** *IC*_50_ (μM) values were determined via the MTT assay. *IC*_50_ values were drug concentrations necessary for 50% inhibition of cell viability. Data are average ± standard deviations of at least three independent experiments. The drug treatment period was 48 h.


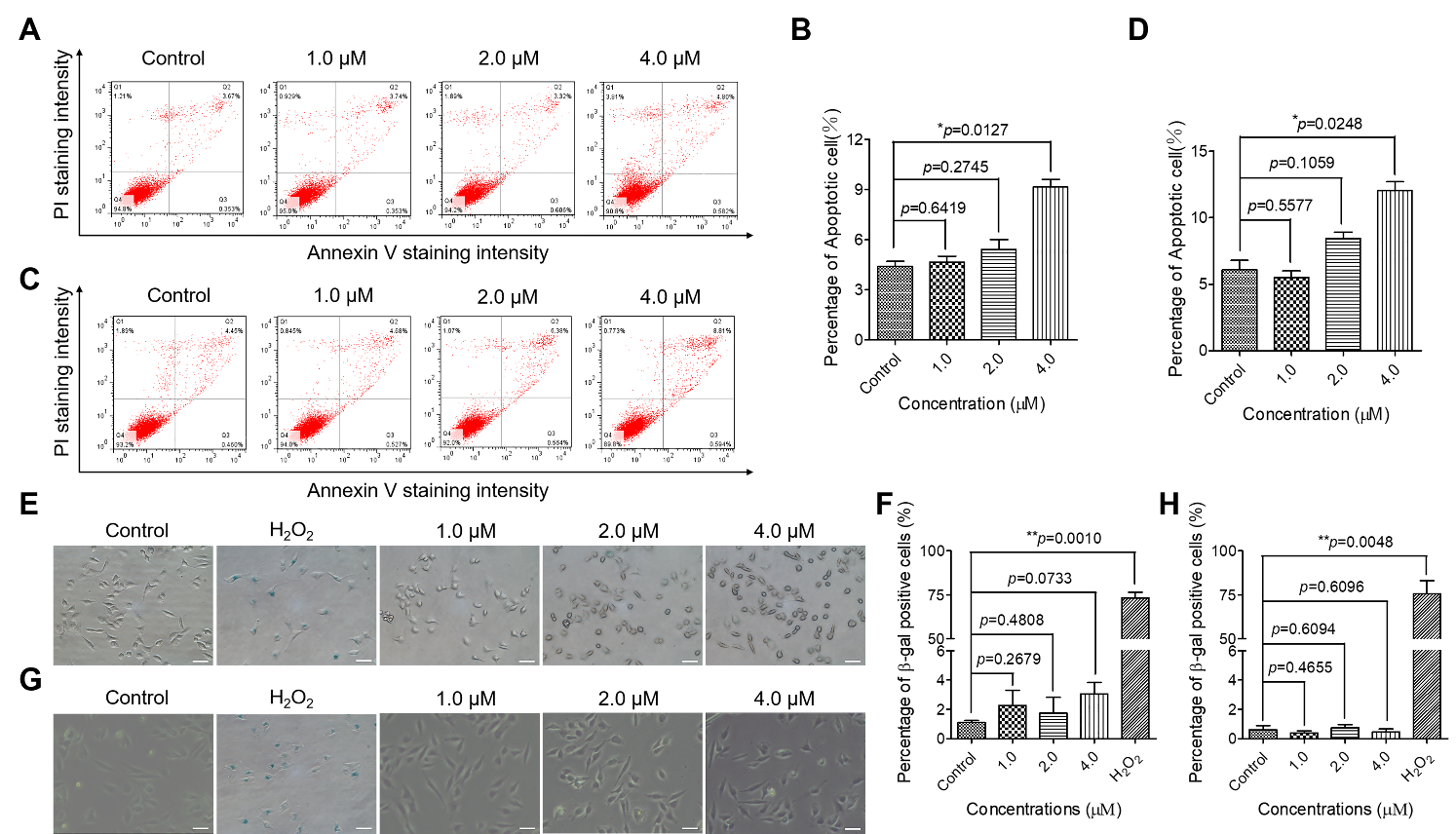


**Supplementary** **Figure 2.** Piperlongumine (PL) treatment did not induce significant cell apoptosis or senescence after the cell migration assay. FACS analysis was performed to detect the apoptotic PC3 **(A)** and DU145 **(C)** cells after the scratch-wound assay with the indicated concentration of PL (1.0µM, 2.0µM or 4.0µM). β-galactosidase staining assay was carried out to assess the senescence of PC3 **(E)** and DU145 **(G)** cells after the scratch-wound assay with the indicated concentration of PL (1.0µM, 2.0µM or 4.0µM). Scale bar, 100 μm. **(B)**, **(D)**, **(F)** and **(H)** were quantification of A, C, E and G, respectively. Values are average ± SD of at least three independent experiments. The statistical significance was calculated using the unpaired student’s two-tailed t-test with the *p*-values (**p* < 0.05, ***p* < 0.01, ****p* <0.001).


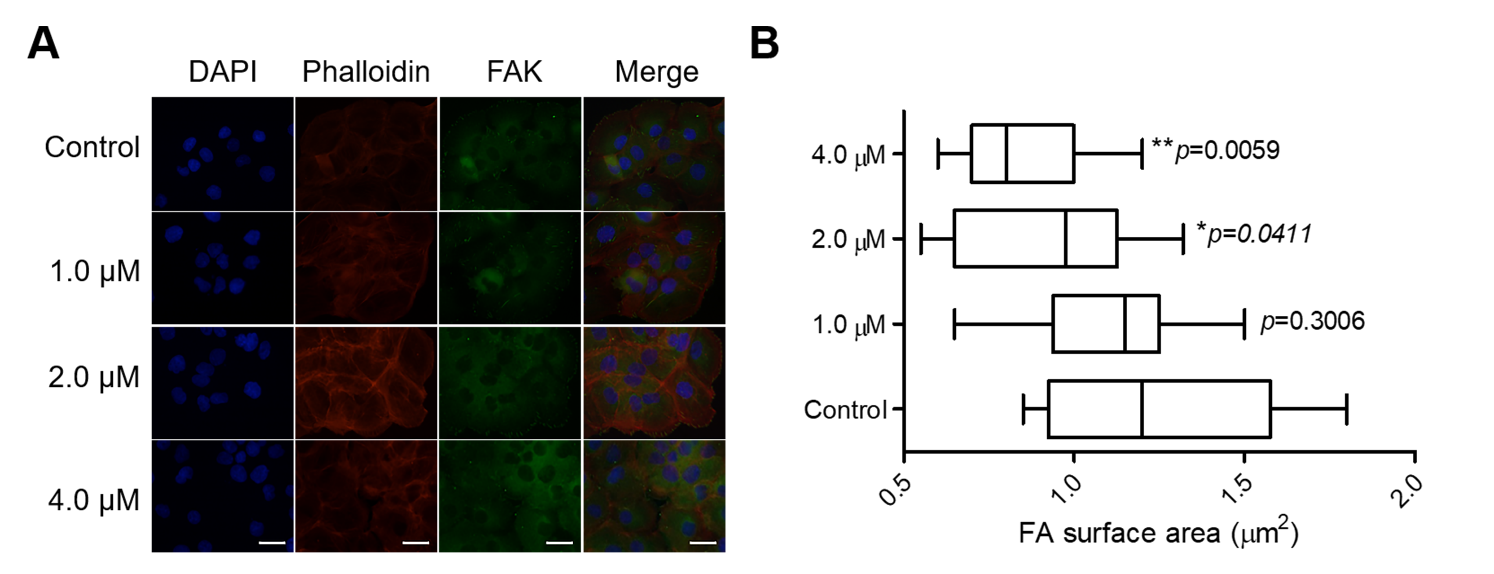


**Supplementary Figure 3.** Piperlongumine (PL) inhibits the expression and distribution of focal adhesion kinase (FAK) in castration-resistant prostate cancer (CRPC) DU145 cells. **(A)**, Representative images of focal adhesion kinase (FAK) in PL-treated and control DU145 cells in immunofluorescence assays. Cells were treated with the indicated concentration of PL for 48h. Cells treated with 0.01% DMSO were used as a control (Control). Antibody to FAK (blue) and phalloidin (red) were used to visualize FAK and F-actin, respectively. Scale bar, 10 μm. **(B)**, Focal adhesion surface area assessed through FAK and phalloidin staining in PL-treated and control DU145 cells. Cells were treated with indicated concentration of PL (1.0µM, 2.0µM or 4.0µM) for 48h. Values are average ± SD of three independent experiments and ≥500 cells were examined in each group. The unpaired student’s two-tailed t-test was used to determine the statistical significance (**p* < 0.05, ***p* < 0.01).

**Supplementary Figure 4.** Piperlongumine (PL) generates reactive oxygen species (ROS) in castration-resistant prostate cancer (CRPC) PC3 and DU145 cells. **(A)**, Intracellular ROS generation in PC3 cells exposed to PL. Cells were treated with 1.0, 2.0 or 4.0 μM PL for 48h and then stained with ROS probe DCFH-DA. NAC pretreatment was carried out at 5 mM for 1h. Representative histogram is shown. **(B)**, Quantification of ROS levels in PC3 cells as determined by DCFH-DA probe. **(C)**, The same as A, except DU145 cells were used. **(D)**, Quantification of ROS levels in DU145 cells as determined by DCFH-DA probe. Values are average ± SD of three independent experiments. The unpaired student’s two-tailed t-test was used to determine the statistical significance (**p* < 0.05, ***p* < 0.01, ****p* < 0.001).

**Supplementary Figure 5.** PL treatment induces cell death in CRPC cells. FACS analysis was performed to detect apoptotic PC3 (A) cells treated with the indicated concentration of PL (1.0, 2.0, or 4.0 µM) over 12 days. β-galactosidase staining assays were carried out to assess the senescence of DU145 (C) cells after PL treatment for 12 days. (B) and (D) represent quantification of A and C, respectively. Values represent the mean ± SD of at least three independent experiments. The statistical significance was calculated using the unpaired student’s two-tailed t-test with the *p*-values (**p* < 0.05, ***p* < 0.01, ****p* <0.001).


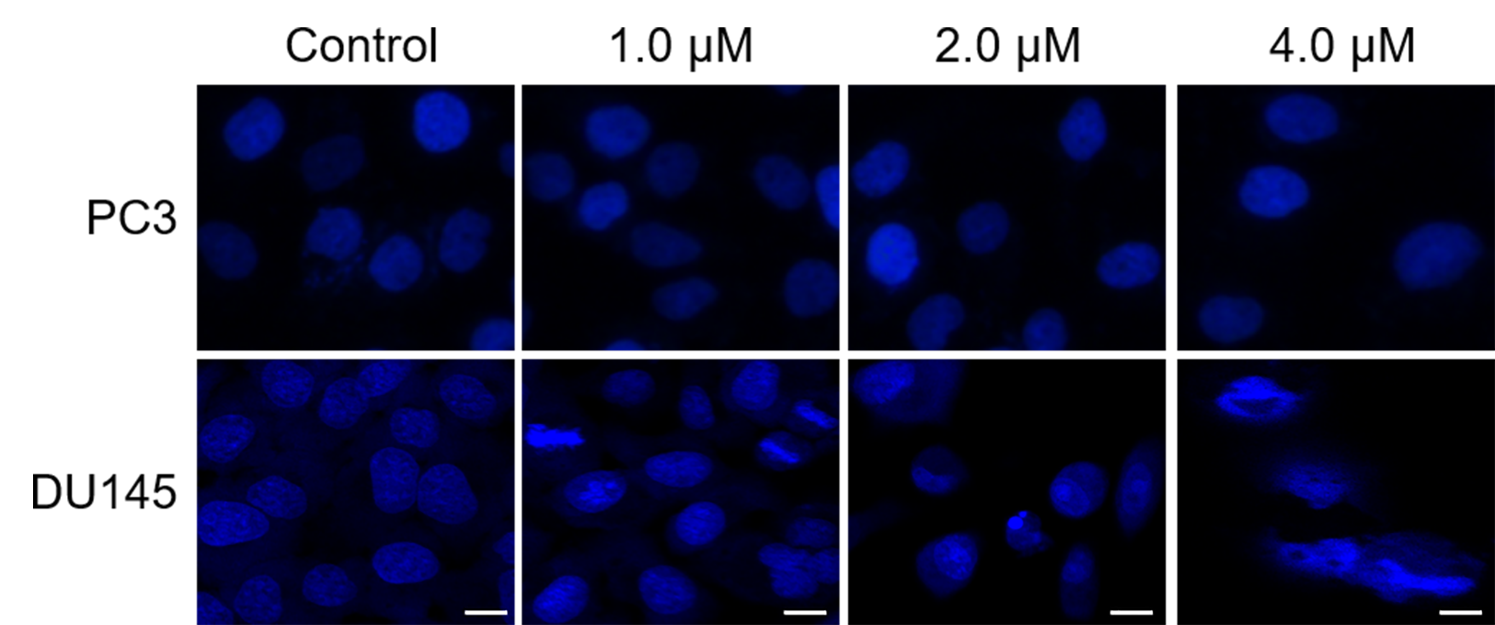


**Supplementary Figure 6.** DAPI stained PC3 and DU145 cells after treatment of PL at indicated concentrations (1.0, 2.0, or 4.0 μM) immediately after long-term cell proliferation of incubation. Scale bar, 100 μm.


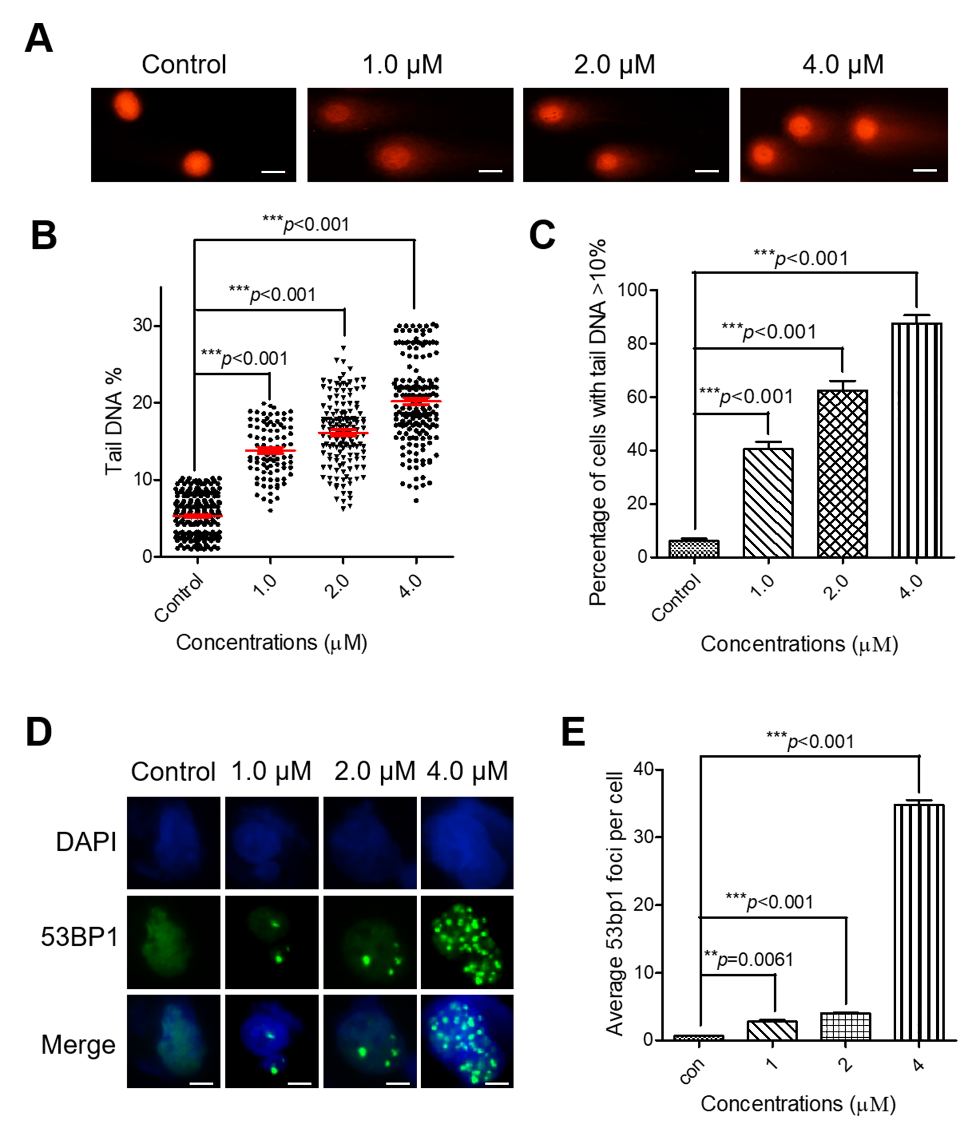


**Supplementary Figure 7.** Piperlongumine (PL) treatment triggers strong DNA damage and provokes intense DNA damage response in prostate cancer (CRPC) DU145 cells. (**A**) The data from comet assay showed that PL treatment triggers strong DNA damage in a concentration-dependent manner. Scale bar, 100 μm. (**B**) and (**C**), the percentages of DNA in the tail for PL treated DU145 cells (**B**) and the percentage of PL treated DU145 with over 10% tail DNA (**C**) were measured. (**D**) The data from immunofluorescence showed that PL treatment provokes intense DNA damage response in a concentration-dependent manner. Scale bar, 10 μm. (**E**) Quantification of (**D**). Cells were treated with indicated concentration of PL (1.0, 2.0, or 4.0 μM) for 48h and ≥200 cells were examined in each group. Values are average ± SD of three independent experiments. The unpaired student’s two-tailed t-test was used to determine the statistical significance (**p* < 0.05, ***p* < 0.01, ****p* < 0.001).


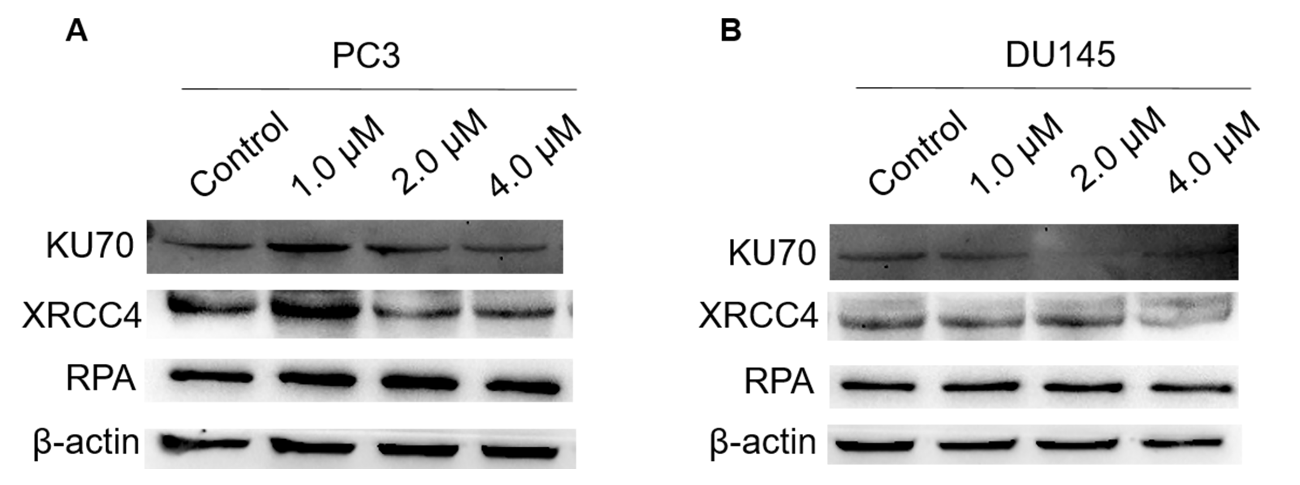


**Supplementary Figure 8.** Western blot assay for evaluating the expression of RPA, XRCC4 and KU70 in indicated concentration PL treated PC3 and DU145 cells. (**A**). The expression of RPA, XRCC4 and KU70 in PC3 cells. (**B**). The expression of RPA, XRCC4 and KU70 in DU145 cells.


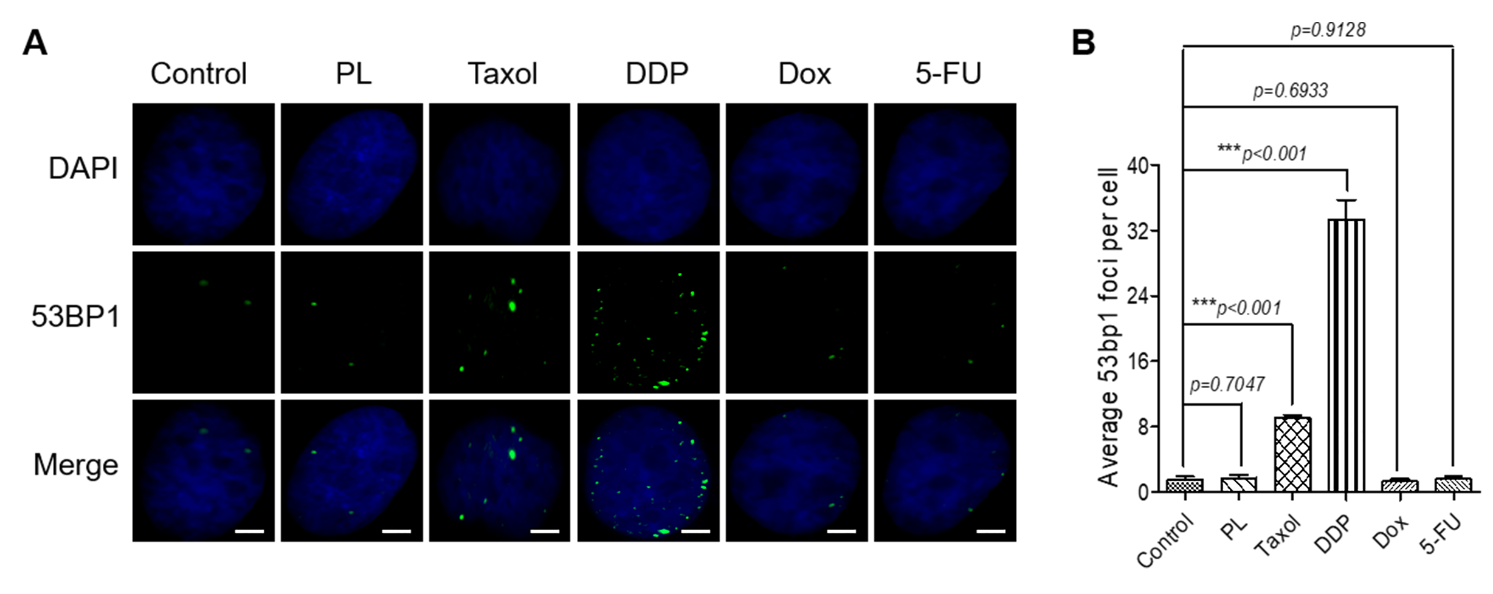


**Supplementary Figure 9.** Immunofluorescence (IF) assay for evaluating the DNA damage. (**A**) LO2 cells were treated with 0.01% DMSO (Control), piperlongumine (PL, 4.0 μM), taxol (4.0 μM), cisplatin (DDP, 4.0 μM), doxorubicin (Dox, 4.0 μM), 5-Fluorouracil (5-FU, 4.0 μM) for 48h. DAPI and 53BP1 was the nucleus dye (blue) and DNA damage marker (green), respectively. Scale bar, 10 μm. (**B**) Quantification of A. The results show the percentage of 53BP1 foci per cell among 200 untreated and treated cells, respectively. The Bar chart of all data represents mean ± SD of three independent experiments, **p* < 0.05, ***p* < 0.01 and ****p* < 0.001.


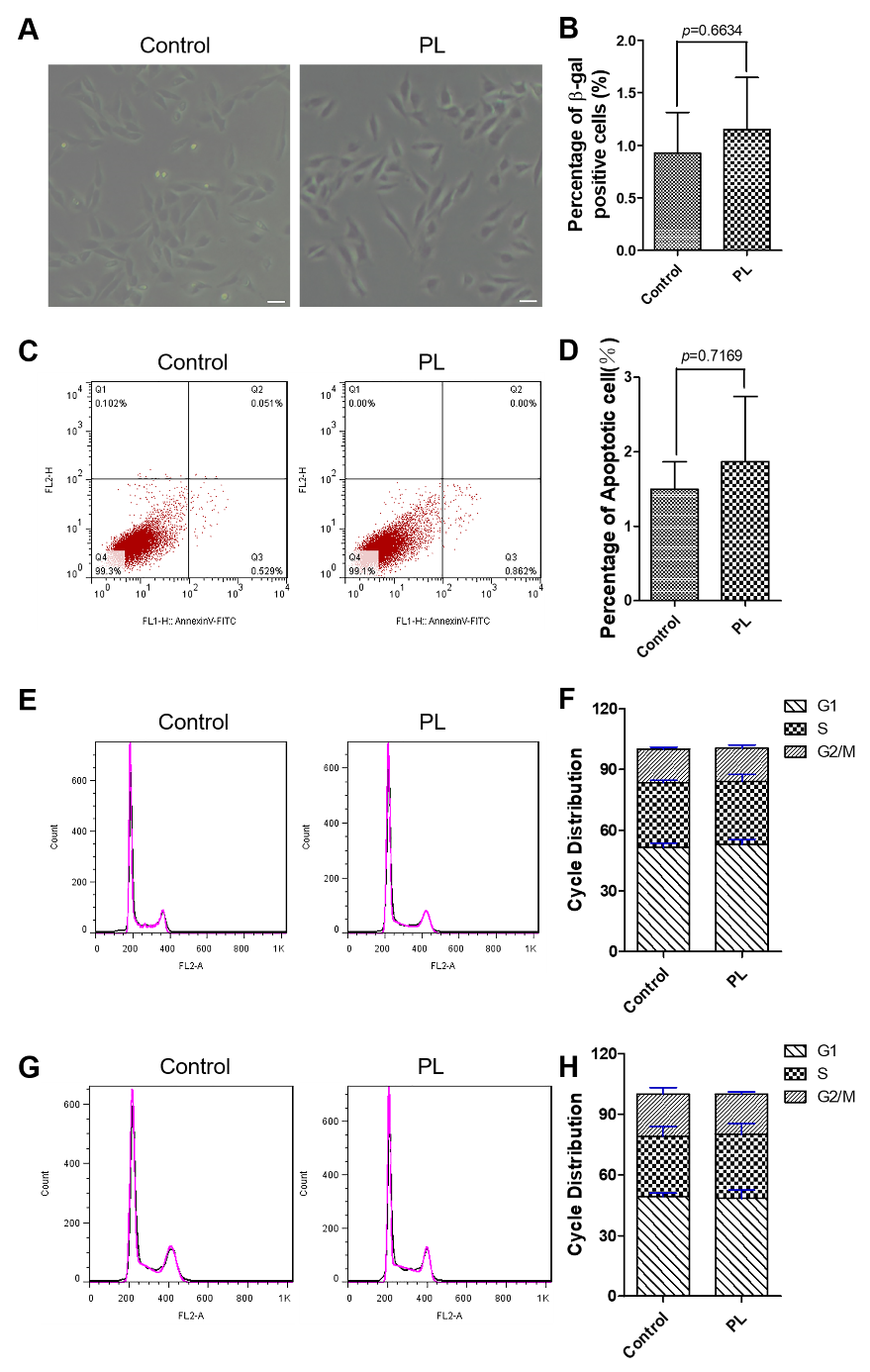


**Supplementary Figure 10.** Piperlongumine (PL) treatment (10µM, 3h) did not induce the senescence, the apoptosis and/or cell arrest of castration-resistant prostate cancer (CRPC) PC3 and DU145 cells. (**A**) Representative results of β-gal staining assay of PL treated PC3 cells. Scale bar, 100 μm. (**B**) Quantitation of the percentage of senescent (β-gal positive) cells in A. (**C**) The percentage of apoptotic cells in PL treated DU145 cells were analyzed by flow cytometry. **(D)** Quantitation of the percentage of apoptotic (Annexin V positive) cells in C. (**E**), Cell cycle arrest of PL treated PC3 cells were detected by FACS. **(F)** Quantitation of E. (**G**) As in E, except DU145 cells were used. **(H)** Quantitation of G. Control indicates cells without PL treatment and values are average ± SD of three independent experiments.


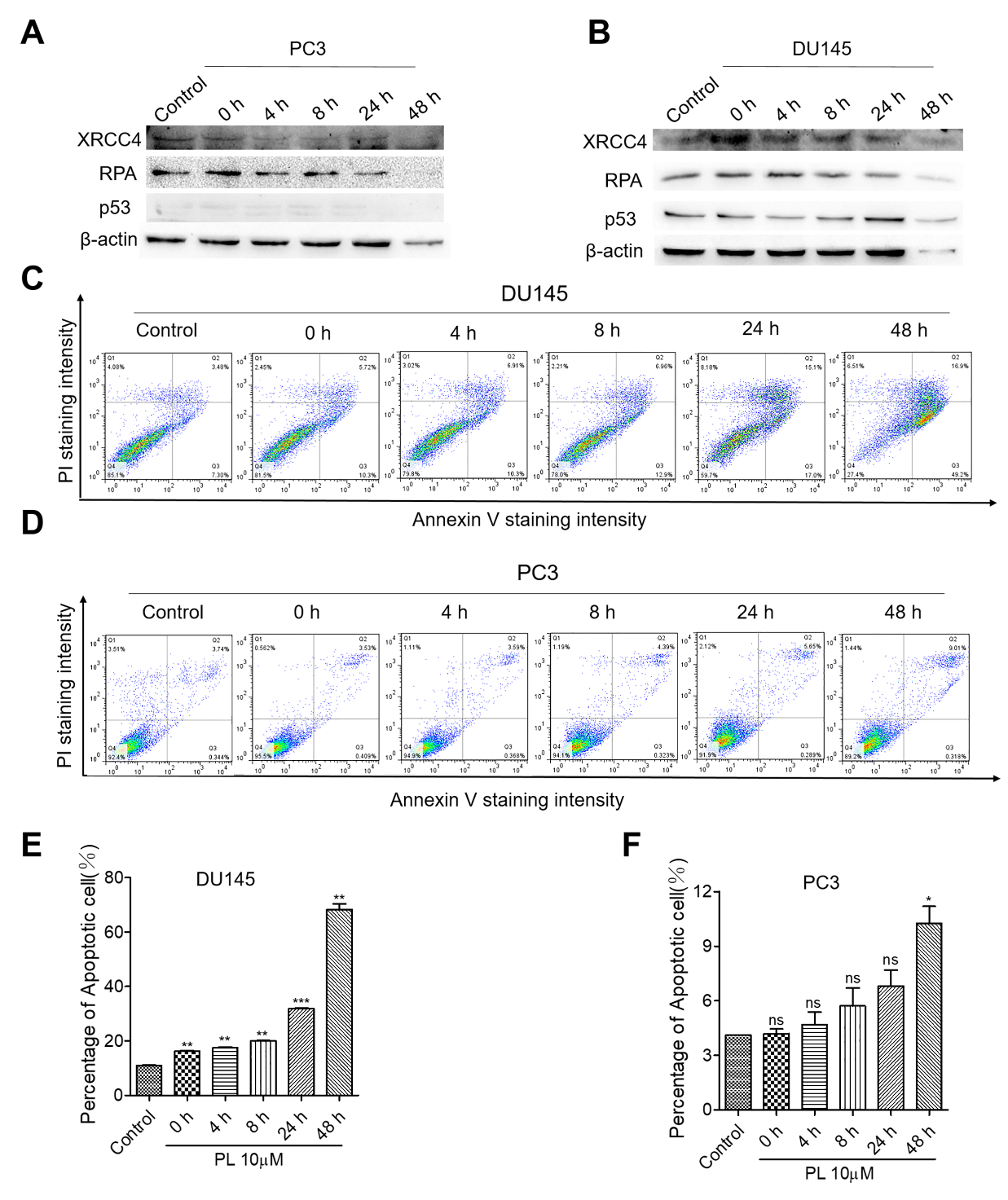


**Supplementary Figure 11.** 10µM PL treatment at indicated time suppressed the process of DNA damage repair in PC3 and DU145 cells and induced apoptosis in DU145 cells. (**A**), (**B**) The expression of p53, RPA and XRCC4 in PC3 and DU145 cells, respectively. (**C**)**, (D)** The percentage of apoptotic cells in PL treated DU145 and PC3 cells were analyzed by flow cytometry, respectively. (**E**)**, (F)** Quantitation of (C) and (D), respectively.
