# Supplementary material for: Piperlongumine inhibits migration and proliferation of castration-resistant prostate cancer cells via triggering persistent DNA damage
Source: BMC Complement Med Ther. 2021 Jul 6;21:195. doi: 10.1186/s12906-021-03369-0 (PMC8261967; doi:10.1186/s12906-021-03369-0)
Supplement: Supplementary file 2 — Additional file 2. Original blot images. [file 12906_2021_3369_MOESM2_ESM.docx]

Original blot images


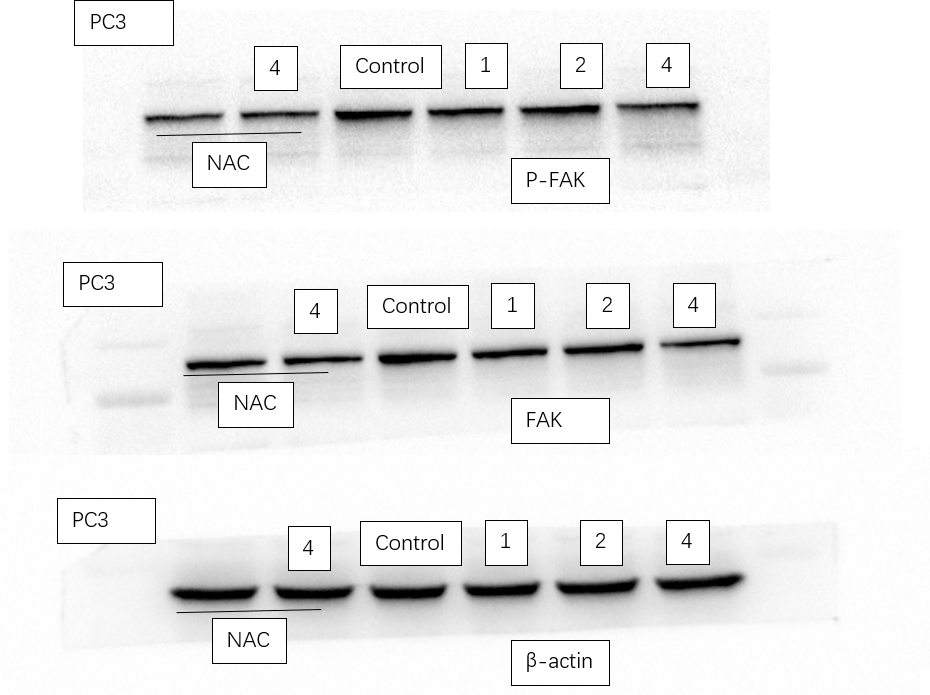


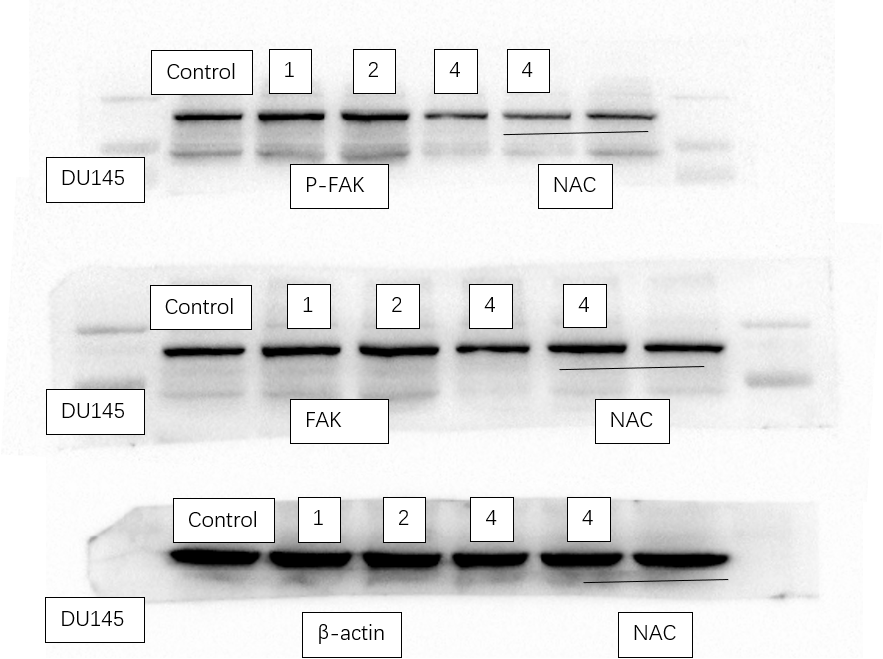


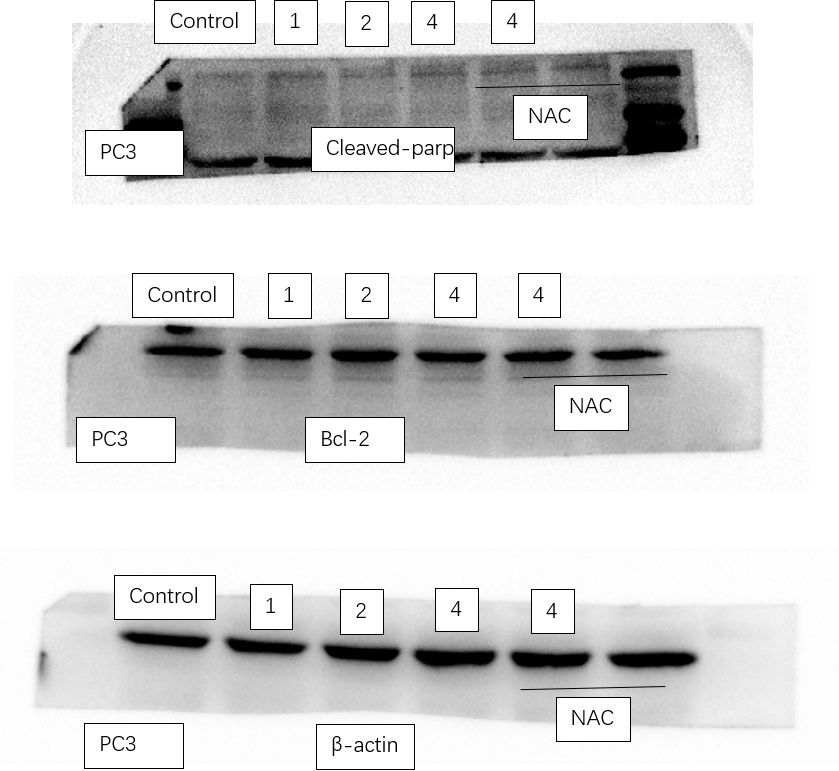


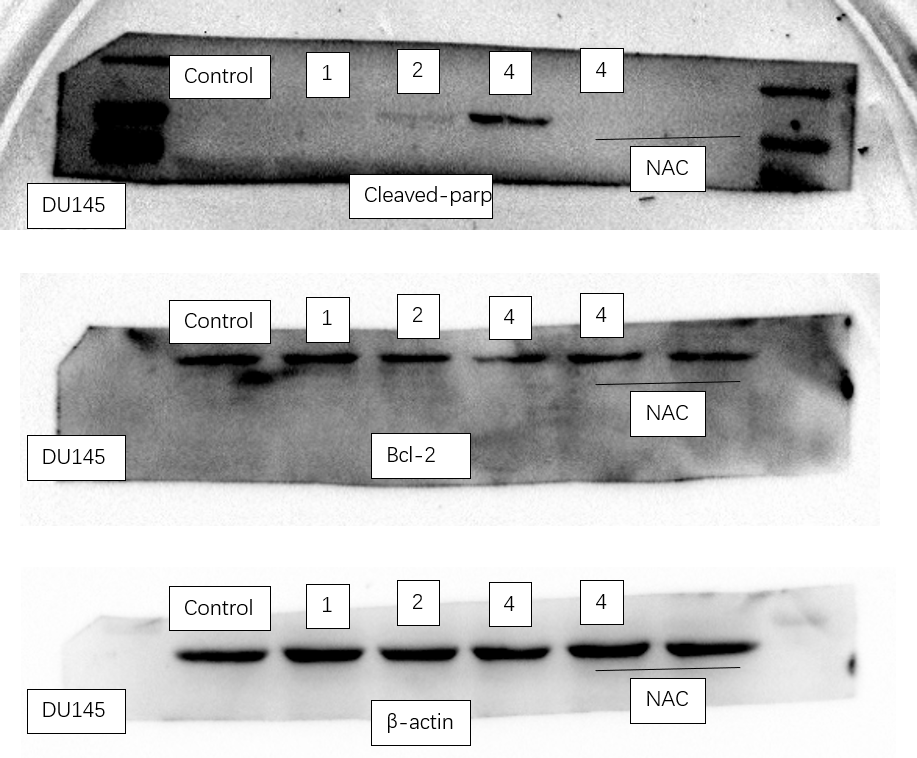


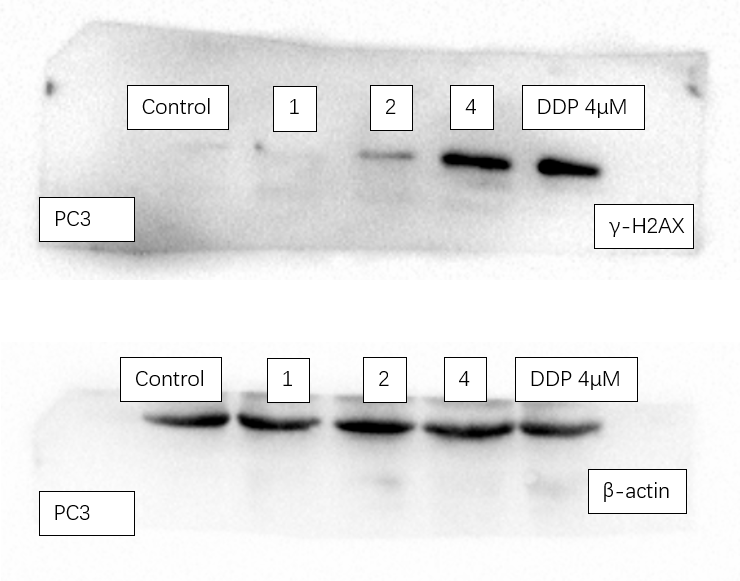


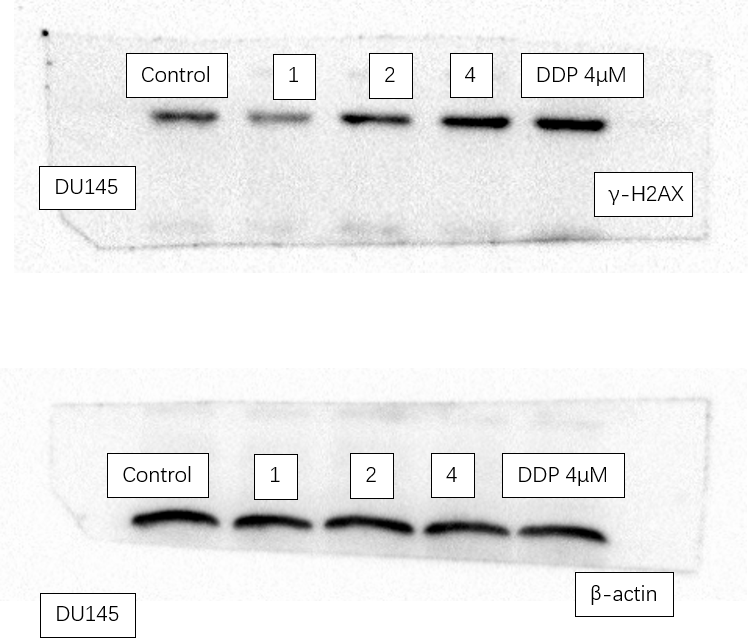


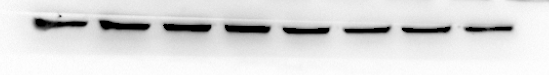

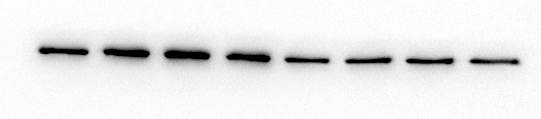

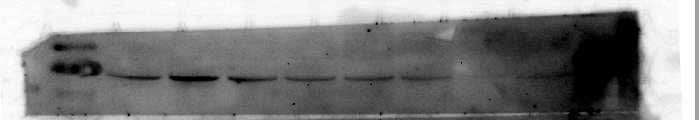

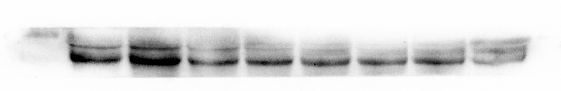


1

4

Control

2

1

4

Control

2

PC3

DU145

XRCC4

KU70

RPA

β-actin


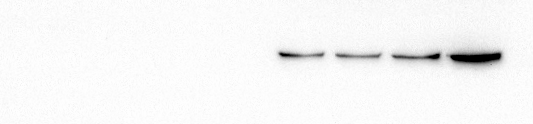


p53


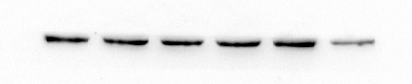

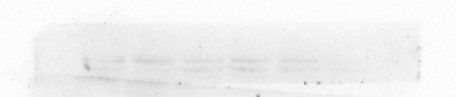

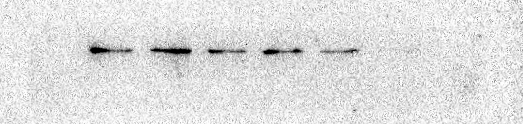

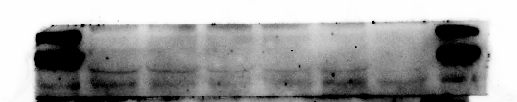


β-actin

RPA

p53

XRCC4

0h

8h

Control

4h

PC3

24h

48h


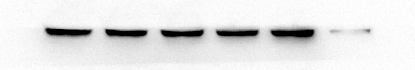

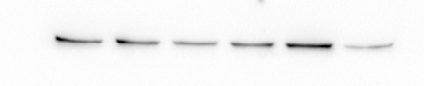

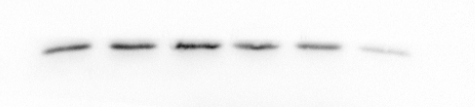

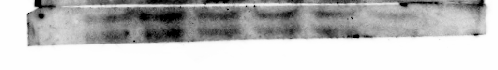


0h

8h

Control

4h

DU145

24h

48h

XRCC4

RPA

p53

β-actin
